# Regulation of MMP9 transcription by ETS1 in immortalized salivary gland epithelial cells of patients with salivary hypofunction and primary Sjögren’s syndrome

**DOI:** 10.1038/s41598-022-18576-z

**Published:** 2022-08-25

**Authors:** Braxton Noll, Farah Bahrani Mougeot, Michael T. Brennan, Jean-Luc C. Mougeot

**Affiliations:** 1 Department of Oral Medicine-Translational Research Laboratory, Carolinas Medical Center, Atrium Health, Charlotte, NC USA; 2 Department of Otolaryngology/Head and Neck Surgery, Wake Forest University School of Medicine, Winston-Salem, NC USA

**Keywords:** Immunology, Molecular biology

## Abstract

Primary Sjögren’s syndrome (pSS) patients exhibit enhanced degradation of the salivary epithelium initially through MMP9 overexpression. We assessed the expression of MMP9 and an associated transcription factor, ETS1, in primary salivary gland epithelial cells (SGECs) and investigated potential regulatory mechanism(s) in immortalized SGECs. SGECs and iSGECs were derived from pSS and/or xerostomic “sicca” patients. siRNA knockdown of ETS1 in iSGECs was performed to determine MMP9 mRNA (qRT-PCR) and protein expression (ELISA). ETS1 binding to MMP9 promoter was assessed by luciferase activity and binding confirmed by mutagenesis and ChIP. Effects of ETS1 overexpression on progenitor and Epithelial-Mesenchymal transition (EMT) associated markers were determined by Western blot. Expression of ETS1 and its phosphorylated form in iSGECs was determined by immunofluorescence microscopy. ETS1 and MMP9 were overexpressed in SGECs of pSS and non-pSS sicca patients with salivary gland lymphocytic infiltration compared to non-pSS sicca patients without infiltration. ETS1 siRNA knockdown reduced both MMP9 mRNA and protein levels. ETS1 overexpression affected the expression of EMT and progenitor cell markers. Lastly, ETS1 bound the MMP9 promoter within the DNA region of −296 bp to −339 bp. ETS1 may impair salivary function through direct transcriptional control of the MMP9 promoter. ETS1 upregulation may also affect other factors involved in repair of the dysfunctional pSS salivary epithelium.

## Introduction

### pSS clinical manifestation and management

Primary Sjögren’s syndrome (pSS) affects approximately 0.4% of the general population with a female to male reported predominance ranging anywhere from 9:1 to 20:1^1–3^_._ Although the disease presents largely within the salivary and lacrimal glands, major systemic complications can occur, which include nephritis, cryoglobulinemic vasculitis, and lymphoma^2,4^. Patients with pSS are at a roughly 13-fold higher risk of developing lymphoma than the general population^5^. The classification of pSS based on disease-defining characteristics is still a matter of debate^6^. Patients with significant salivary and/or lacrimal hypofunction characterized by the presence of anti-SSA (Anti-Ro52/60/SSA Sjögren’s syndrome antigen A) autoantibodies in serum and/or substantial salivary infiltration by inflammatory cells are designated as pSS^7^. The cut-off for substantial infiltration by inflammatory cells which defines pSS is a focus score (FS) of 1 corresponding to the presence of at least one focus (focal accumulation of cells) with at least 50 inflammatory cells per 4 mm^2^ tissue area^6^. Patients with salivary hypofunction, exhibiting limited or no immune cell infiltration, and characterized by the absence of anti-SSA auto-antibodies in the salivary and lacrimal glands are designated as “sicca” patients” according to the current ACR/EULAR criteria^7^. pSS usually presents in mid-life (40–50 years of age) and generally evolves over several years^2,8^.

Currently, pSS is treated mainly symptomatically through the administration of immunosuppressants in conjunction with compounds used to stimulate fluid secretion^9^. Even with the advent of antibody targeted therapies, none have yet to be adopted into mainstream practice^9^. In 2016, the American College of Rheumatology-European League Against Rheumatism (ACR-EULAR) joint organization re-defined the symptoms and clinical criteria for pSS, increasing the emphasis of anti-SSA positive serology^6^. Roughly 24% of pSS patients remain autoantibody negative^10^. The relative ineffectiveness of biological therapies such as rituximab for pSS combined with a lack of ubiquitous autoantibody expression, highlights other non-immunologic aspects contributing to disease onset and pathogenesis^8–10^.

### Non-immunologic early onset and pathogenesis of pSS

pSS pathology has been described as an “autoimmune epithelitis,” affecting epithelia both systemically (e.g., interstitial nephritis, glomerulonephritis, primary biliary cirrhosis, lymphocytic interstitial pneumonia) and locally within the salivary and lacrimal glands^11^. A loss of salivary epithelial homeostasis is demonstrated in pSS pathogenesis where disruptions to epithelial architecture are commonly observed^12^. Changes to salivary and lacrimal functions were shown to occur during the “pre-immune” phase of pSS mouse models prior to autoimmune development and lymphocytic infiltration^13–15^.

The extracellular matrix (ECM) is the primary structure essential to epithelial homeostasis and maintaining salivary gland epithelial cell function^16,17^. Degradation of ECM structures by proteolysis and breakdown of topological epithelial integrity are prominent traits early in pSS pathophysiology^18^. Interruptions in ECM-cell connections trigger various processes, such as anoikis (a form of ECM detachment-associated apoptosis) or an Epithelial-Mesenchymal Transition (EMT) response in the salivary epithelium^19,20^. Detachment of acinar cells from the ECM disrupts homeostatic communication, hampering development and survival signaling among neighboring cells^12^.

### Role of MMP9 in pSS pathobiology

In pSS patients’ salivary glands, the ECM is under perpetual degradation and remodeling by matrix metalloproteinases (MMPs), including MMP9^21^. MMP9, also known as Gelatinase B, is a zinc-dependent endopeptidase, capable of degrading tight junction proteins and protein components located in multiple layers of the ECM (i.e., collagen IV, V, XI; laminin; elastin; aggrecan)^22,23^. MMP9 is overexpressed by the salivary epithelium in pSS without proximity to immune cell infiltration in glandular tissue^24–26^. In pSS patients, acinar and ductal cells have been shown to be responsible for local MMP9 secretion. Additionally, MMP9 glandular expression and activity are highly correlated with the degree and severity of salivary gland damage and functional changes^21,25^. The mechanisms triggering and governing the overexpression of MMP9 in pSS are poorly understood.

### ETS1 and LEF1 as potential regulators of MMP9 expression in pSS

We have previously shown the overexpression of two transcription factors, namely V-Ets Avian Erythroblastosis Virus E26 Oncogene Homolog 1 (ETS1) and Lymphoid Enhancer-Binding Factor 1 (LEF1) in labial salivary glands (LSGs) of pSS patients^27,28^. In pSS patients, ETS1 and LEF1 were co-overexpressed with MMP9 in the glandular epithelium without proximity to lymphocytic (CD4^+^) infiltrates, demonstrating their localized dysregulation^28^. ETS1 has functions in multiple critical pathways implicated in pSS pathogenesis (i.e., ERK/MAPK, Ras-MAPK, p-38 and Ca^2+^ signaling), impacting cell differentiation/development, cytokine production, hematopoiesis, and EMT^29–31^. ETS1 activity is primarily attributed to its hallmark ETS binding site (EBS) 5′-GGA(A/T)-3′ motif, although cooperative actions with other factors (e.g., AP-1, RUNX, PAX3/5) enables binding to sites deviating from its core recognition sequence^29,32^ . LEF1 is involved in the Wnt signaling mediated by CTNNB1 (β-catenin) pathway. LEF1 also acts as a transcriptional activator that targets genes involved in EMT, cell migration, stem cell maintenance, and differentiation^30,33^. LEF1 is an architectural transcription factor, which facilitates bending and loop formation of DNA into higher order structures with distant transcription factors such as ETS1, thereby regulating T-cell receptor expression through a remote enhancer site^34^. Overall, ETS1 and LEF1 are critical drivers of EMT, regulating MMP9 in a variety of epithelial cell types^33,35,36^. However, their role in regulating MMP9 expression in pSS salivary gland epithelium has not been determined^33,35,36^.

Here, we establish the role of ETS1 as a direct regulator of MMP9 mRNA expression in our cultured primary salivary gland epithelial cells (SGECs) from labial salivary gland biopsies of sicca and pSS patients. Further, using immortalized SGEC (iSGEC) lines derived from one pSS and one sicca patient^37^, and two salivary gland cell lines (SGCLs) of oral cancer origin, we demonstrate the regulation of MMP9 by ETS1 through direct promoter binding. We also determined the downstream effects of ETS1 both in pSS and non-pSS derived iSGECs regarding the expression of progenitor cell and EMT protein markers.

## Experimental procedures

### Salivary gland cell lines: A253, HMC-3A, iSGEC-nSS2, and iSGEC-pSS1

The salivary gland cancer cell line model, A253, originates from a submaxillary salivary gland epidermoid carcinoma and were cultured per ATCC’s recommended protocol. HMC-3A cells were derived from a mucoepidermoid carcinoma of the left hard palate and were provided as a generous gift from Dr. Jacques E. Nor, DDS and Kristy Warner (University of Michigan School of Dentistry, Ann Arbor, MI, USA) and cultured as outlined by Warner et al.^38^ Immortalized salivary gland epithelial cell lines (iSGEC-nSS2 and iSGEC-pSS1) were previously generated in our laboratory^37^. iSGEC-nSS2 and iSGEC-pSS1 immortalized cell lines were derived from a non-pSS “sicca” female patient with salivary hypofunction and a focus score (FS) of 0.16 and a female pSS patient with salivary hypofunction and FS = 1.8, respectively. iSGEC cultures were grown in serum-free Epi-life Basal media (Gibco) without HKGS for 24 h prior to experimentation. Detailed procedures are described in Supplementary Methods.

### Primary culture of salivary gland epithelial cells

LSG biopsies were collected from xerostomic patients undergoing routine diagnostic evaluation for glandular lymphocytic infiltrates^6^. Clinical information for patient groups (i.e., non-pSS (FS = 0), non-pSS (0 < FS < 1), and pSS (FS ≥ 1)) is presented in Table 1. The entire study and all associated experimental procedures were approved by the Atrium Health Institutional Review Board (Charlotte, NC, USA). All experiments were performed in accordance with the Helsinki declaration and following all relevant guidelines and regulatory practices. After obtaining informed consent for each participant, salivary gland tissue was acquired and primary cultures were carried out as previously described^39,40^. SGEC cultures were maintained in serum-free Epi-life Basal media (Gibco) with HKGS (1x) (Gibco) at 37 °C and 5% CO_2_. Media were replenished every three days and cells passaged 3–5 × were used for mRNA extraction. Detailed SGEC culture protocols are in Supplementary Methods.

**Table 1:** Demographics and clinical features of xerostomic patients from whom primary SGEC cultures were derived.

	FS = 0	0 < FS < 1	FS ≥ 1
**Demographics**			
Subjects (N = 23)	n = 5	n = 9	n = 9
Age	31–69, AVG = 44.4, SD +/−14.3	38–78, AVG = 59.1, SD +/−11.7	36–70, AVG = 54.6, SD +/− 9.9
Sex	5/5 Female	9/9 Female	9/9 Female
**Clinical features**			
Focus Score	0	0.16–0.9	1.0–3.52
Anti-Ro (SSA) positive	0/5	0/9	2/6*
Unstimulated salivary flow (Average mL/min)	0.1272	0.08911	0.07473^$^
Schirmer's positive	2/5	3/9^#^	2/9^&^

Salivary gland epithelial cell cultures (SGECs) were derived individually (not pooled) from a total of N = 23 patients receiving labial salivary gland biopsies as part of the routine diagnostic approach following the 2016 ACR-EULAR classification criteria. Patients were subdivided into three separate groups by focus score (FS) for comparisons (FS = 0, 0 < FS < 1, and FS ≥ 1). All SGEC cultures in this study were derived from female patients and treated identically following the same procedure. The presence of Anti-Ro (SSA) serum was not determined/ tested for in three FS ≥ 1 patients. Of the remaining six FS ≥ 1 patients, only two were positive ( +) for the presence of serum Anti-Ro (SSA). ^$^One patient with focus score of 1.3 but negative Schirmer’s test, and unstimulated salivary flow of 0.332 mL/min was excluded from average calculation. Status was unavailable, due to lack of test or non-consent: *three patients; ^#^three patients; ^&^one patient.

### Generation of stable clones

HMC-3A cells (3 × 10^5^ cell/well) were seeded in 6-well plates and allowed to adhere for 24 h prior to transfection. pCMV3-Empty or pCMV3-ETS1 from SinoBiological (500 ng) were used for transfection (1.5 µl of Lipofectamine 3000/well) (ThermoFisher). After 72 h, cells were treated with 2xLD_50_ concentration of hygromycin (Enzo Life Sciences) for initial selection process and then reduced to 1xLD_50_ for remaining clonal selection.

### MMP9 promoter plasmid constructs

The online NCBI primer-blast tool was used to design primers for MMP9 promoter luciferase-reporter plasmids (NCBI Entrez Gene: 4318) (Supplementary Table 1). The −923 bp to + 18 bp of the MMP9 proximal promoter was amplified by PCR using Phusion Polymerase (ThermoFisher). Restriction sites were incorporated into the forward (*mluI*) and reverse (*xhol*) primers of the initial −923 bp to + 18 bp promoter section for insertion into a pGL3-basic *renilla* luciferase reporter plasmid (Promega) upstream of the luc + gene. Remaining 5′-deletion fragments (−439 bp MMP9), (−366 bp MMP9), (−216 bp MMP9) were generated by PCR and cloned into the pGL3-basic luciferase reporter plasmid.

### MMP9 promoter luciferase assay

SGCLs were seeded at approximately 70–80% confluency in 12-well plates 24 h prior to transfection. Lipofectamine 3000 (ThermoFisher) was used for transfection following the manufacturer’s recommended protocol. In each experiment, 300 ng of either the control vector (pGL3-basic) or MMP9 proximal promotor pGL3 vector was co-transfected with 30 ng pRL-TK (*renilla* luciferase plasmid) under the control of a thymidine kinase promoter using Dual Luciferase Assay system (Promega). Relative luciferase units (RLU) were calculated by the normalization of pGL3 luciferase activity to the co-transfected control plasmid (pRL-TK (Promega).

### Promoter truncates and site-directed mutagenesis

Preliminary putative ETS1 transcription factor binding sites on MMP9 proximal promoter region were determined using online tool ALGEN-PROMO^41,42^. Mutations of the five consensus ETS1 (5′-GGA/T-3′) binding sites (EBS) between −216 to −366 were generated by site-directed mutagenesis using primers described in Supplementary Table 1. Detailed methods are in Supplementary Methods.

### siRNA knockdown and transient transfection

Native expression of ETS1 was knocked down using SMARTpool siRNAs corresponding to a mixture of 4 siRNAs targeting ETS1 (Horizon) and Lipofectamine 3000 (ThermoFisher). The siGENOME non-targeting siRNA pool #2 (Horizon) served as negative control. Relative expression knockdown was calculated using average ΔCT of three independent experiments including non-targeting siRNA as control, from which relative ΔCT values were generated. Cells were harvested after 48 h for mRNA isolation or 72 h for protein isolation. Transient transfection of iSGECs with overexpression plasmids was performed similarly with the addition of 500 ng of plasmid DNA added per well.

### Quantitative real-time PCR

Total RNA was isolated from cells using the RNeasy mini kit (Qiagen) following the manufacturer’s protocol. RNA (500 ng) from each sample was reverse transcribed using TAKARA MMLv reverse transcriptase kit following the provider’s instruction. Random Hexamers were purchased from IDT. dNTPs were purchased from Promega. Levels of ETS1, LEF1, and MMP9 were expressed as relative to GAPDH based on ΔCt method. qRT-PCR primers are listed in Supplementary Table 1.

### Western blot

Levels of target proteins in whole cell lysates of iSGECs, A253, and HMC-3A post-transfection were assessed by Western blot. Detailed methods are presented in Supplementary Methods. Antibodies and concentrations are listed in Supplementary Table 2.

### ELISA

MMP9 secretion into media was measured by QuickZYME MMP9 (Quicktime Biosciences). MMP9 (active and inactive) quantification was normalized to total cellular protein. Experimental relative fold changes were determined by comparison to the non-targeting siRNA control. Detailed ELISA methods are described in Supplementary Methods.

### Chromatin immunoprecipitation (CHIP) assays

ChIP assays were performed using the EpiQuick Tissue Chromatin Immunoprecipitation Kit per manufacturers’ instructions. Nuclear extracts were immunoprecipitated with antibodies targeting ETS1 (SCBT) or normal mouse IgG and subjected to qPCR with primers targeting the MMP9 promoter region from −197 bp to −421 bp (Supplementary Table 1). Experimental samples and controls (mouse IgG) were normalized to 5% of the initial input of non-immunoprecipitated DNA. Detailed ChIP protocol is described in Supplementary Methods.

### Detection of protein expression in iSGECs by immunofluorescence (IF)

Untreated iSGECs were cultured on 8-well chambered slides (ThermoFisher) coated with gelatin (0.2%) for 24–48 h prior to fixation with 4% PFA. Dilutions for primary and secondary antibodies are listed in Supplementary Table 2. Detailed IF methods are described in Supplementary Methods.

### Statistical analysis

Correlation of mRNA expression among SGECs was determined using Spearman’s rank correlation (α = 0.05). Comparisons were performed using Mann–Whitney U-test to determine significant differences between groups (α = 0.05). Data are presented as mean +/− standard deviation (SD). Experiments were performed with a minimum of three biological replicates using 3 wells per condition. Statistical analyses were conducted using Graphpad Prism 9.2.

## Results

The experimental approach and design for characterizing the ETS1 mediated regulation of MMP9 are outlined in Supplementary Fig. 1.

### Differential expression of ETS1, LEF1 and MMP9 in cultures of primary and immortalized salivary gland epithelial cells derived from patients with salivary hypofunction

**Figure 1:**
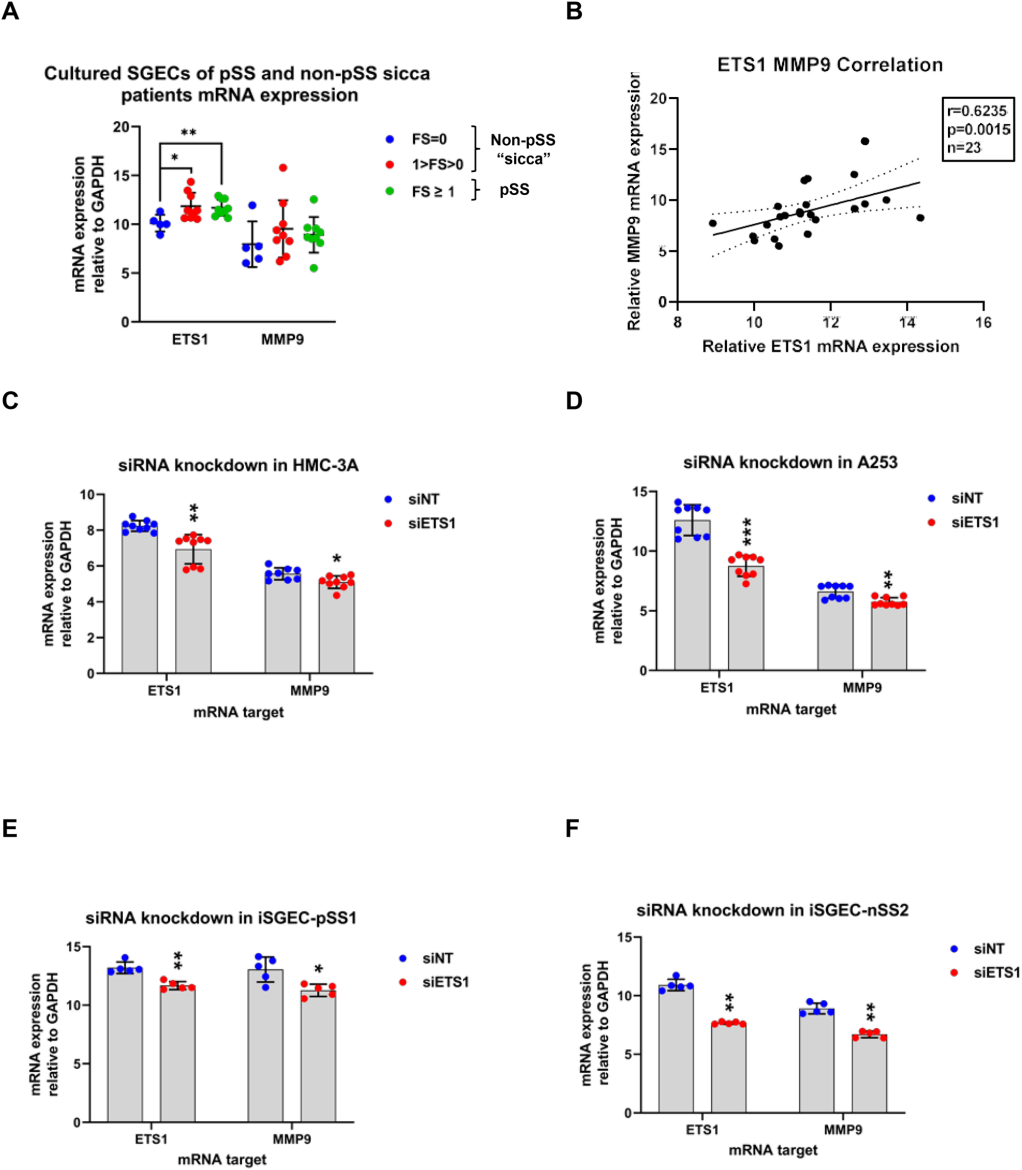
Effects of ETS1 siRNA mediated knock down on MMP9 mRNA expression in salivary gland derived cell lines. Explant SGEC cultures were obtained from pSS(FS ≥ 1) (n = 9), non-pSS(10) (n = 9), and non-pSS(FS = 0) n = 5) patients. (**A**) qRT-PCR expression of ETS1 and MMP9 in primary SGEs was normalized to GAPDH and compared to FS = 0 by Mann–Whitney U-test. Error bars represent mean (+/−) standard deviation (SD). SGECs of pSS (FS ≥ 1) over express both ETS1 (p = 0.007) and MMP9 (p > 0.05) compared to non-pSS (FS = 0) patients. LikepSS (FS ≥ 1), non-pSS (1 < FS > 0) SGECs over expressed ETS1(p = 0.0120) compared to the FS = 0 group. (**B**) Spearman's rank correlation coefficient (*rho*) was used to assess correlations among ETS1 and MMP9 mRNA expression in primary SGECs (n = 23). ETS1 expression correlated with MMP9 (r = 0.6235, p = 0.0015, n = 23) (**B**). mRNA expression of ETS1 and MMP9 was determined in the salivary gland derived cell linesHMC-3A (**C**), A253 (**D**), iSGEC-pSS1 (**E**), and iSGEC-nSS2 (**F**), 48 h post-transfection with siRNA targeting ETS1byqRT-PCR. Knockdown of ETS1 (siETS1) were compared to a non-targeting (siNT) control. qRT-PCR mRNA expression was normalized to the housekeeping gene GAPDH. Knockdown of ETS1 consistently led to a reduction of MMP9 mRNA expression. Data are presented as the mean +/− standard deviation (SD) (n = 6): *p < 0.05, **p < 0.01, ***p < 0.001.

To determine the relationship between MMP9, ETS1, and LEF1, we cultured primary SGECs of non-pSS (n = 14; FS = 0, 0 < FS < 1) and pSS (n = 9; FS ≥ 1) patients. ETS1 was overexpressed in SGECs from pSS (FS ≥ 1), and non-pSS (0 < FS < 1) groups compared to the non-pSS FS = 0 group (p < 0.05). MMP9 was expressed higher (p > 0.05) in pSS (FS ≥ 1) patients, which is in-line with a previous study assessing MMP9 levels in pSS minor salivary gland biopsies (Fig. 1A)^28^. ETS1 and MMP9 mRNA expression displayed a significant correlation in cultured SGECs (p = 0.0015, r = 0.6235) (Fig. 1B).

To further examine MMP9 regulation by ETS1, SMARTPool siRNAs targeting ETS1 were transfected into two iSGECs cell lines, iSGEC-nSS2 (FS = 0.16) and iSGEC-pSS1 (FS = 1.8), and two salivary gland cancer derived cell lines, HMC-3A and A253 (Fig. 1C–F). In all cell lines tested, MMP9 mRNA expression was significantly decreased when ETS1 mRNA expression was reduced.

### Reduction of intracellular MMP9 expression and secreted MMP9 by siRNA knockdown of ETS1

**Figure 2:**
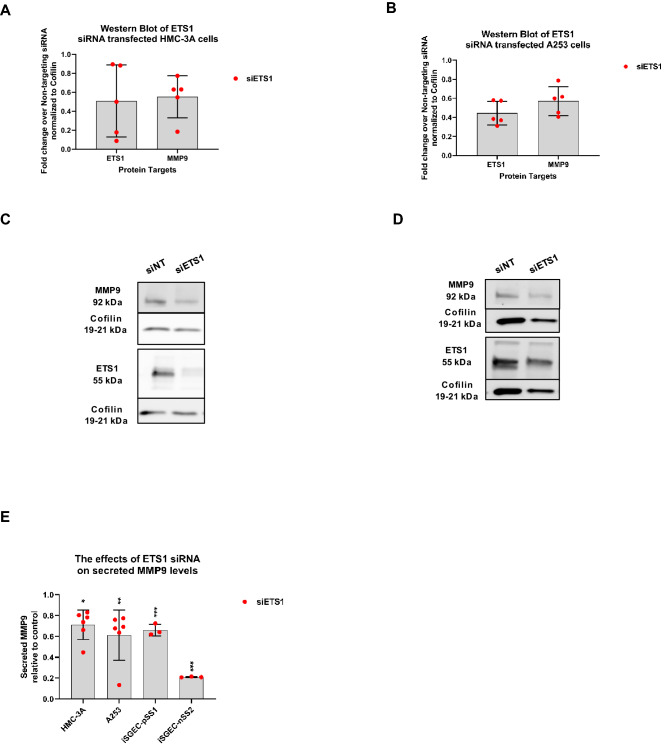
Effects ofETS1 siRNA-mediated knockdown on MMP9 protein expression in salivary gland derived cell lines. Semi-quantitative(densitometric) Western blotanalysis(**A/B**)and representative Western blots(**C/D**) of siRNA knockdown experimentsareshown. ETS1andMMP9 protein levels were determined 72hrspost-transfection with siRNA targetingETS1 (siETS1), relative to non-targetingsiRNA (siNT) (control)in HMC-3A(**A/C**)and A253(**B/D**)whole cell lysates. MMP9 protein levels were reduced in bothA253 and HMC-3A cells when transfected with 3 A cells when transfected with siETS1. Equal protein amounts were loaded into each lane and respective target normalized to cofilin protein expression. Western blot sections corresponding to either (ETS1 and Cofilin) or (MMP9 and Cofilin) were acquired from the same gel/blot, percell line, and processed identically from the same replicate sample, if possible. Loading controls and target proteins of the same gel/blotare imaged separately for optimized exposure requirements among the loading control *vs*. target protein (s). The effectsofETS1(siETS1) siRNA knockdown onMMP9 protein secretion into culture media were also determined (**E**).Total MMP9 (active and inactive) was measured by ELISA and presented as fold-decrease of the non-targeting siRNA (siNT) control. InSGCLs HMC-3A (n = 6) and A253 (n = 6), there were significant reductions in total MMP9 protein supernatant levels after siRNA knockdown of ETS1(**E**). Total MMP9 in the cell culture supernatant of iSGEC-pSS1 (n = 3) and iSGEC-nSS2 (n = 3) was significantly reduced by ETS1 knockdown (**E**). Mann–Whitney U-test was used to determine significant differences among the control (siNT) and siETS1. Results are presented as mean +/− standard deviation (SD). (***p < 0.001), (**p < 0.01), (*p < 0.05).

To further investigate the regulation of MMP9 by ETS1, we evaluated the effects of ETS1 siRNA knockdown on MMP9 protein levels after 72 h in whole cell lysate of HMC-3A and A253 cells (Fig. 2A–D). Western blot analysis showed MMP9 protein was decreased in both HMC-3A and A253 cells by ETS1. Secreted MMP9 protein levels were assessed by ELISA (Fig. 2E). ETS1 inhibition led to a significant decrease in secreted total MMP9 of all four cell lines (p < 0.05).

### Identification of the ETS1 responsive region(s) within MMP9 promoter

**Figure 3:**
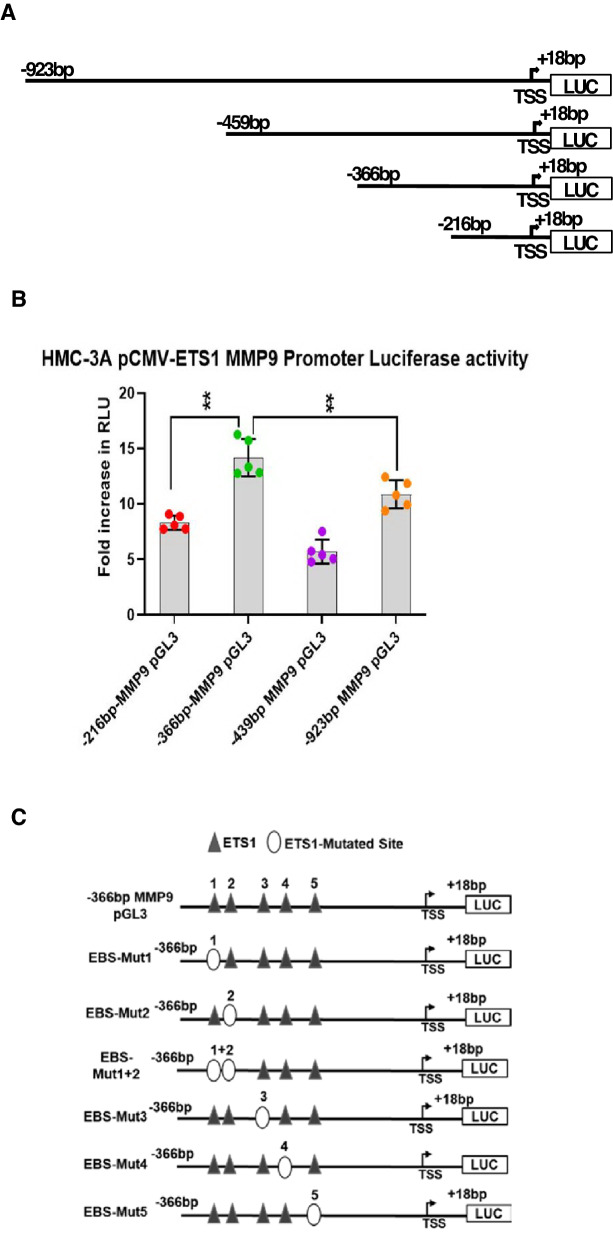

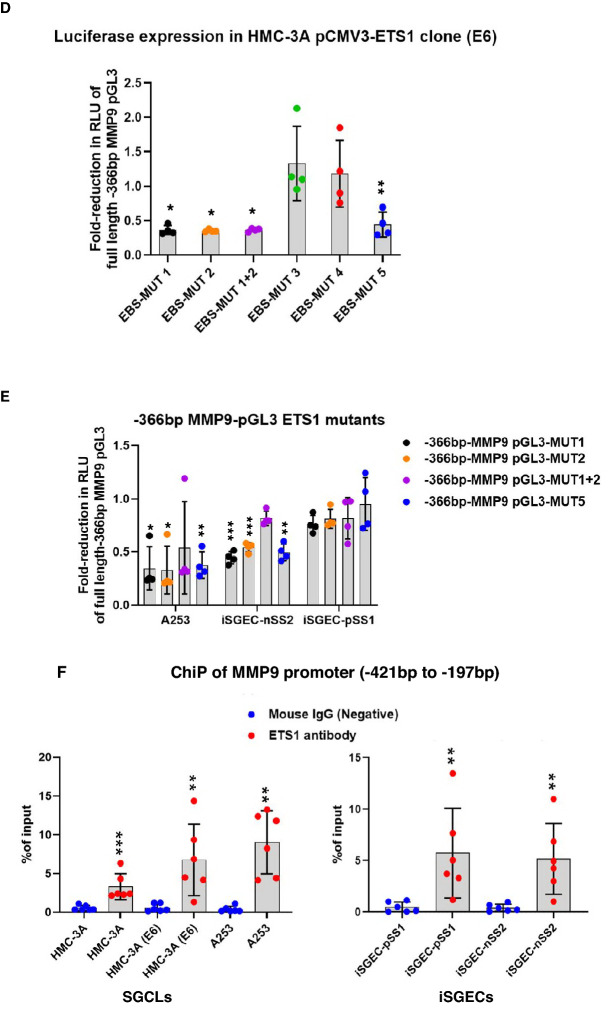
ETS1 binding and regulation of MMP9 promoter transcription in HMC-3A, A253, and iSGECs. (**A**) HMC-3A (E6) and control HMC-3A (EM) were transiently transfected with either 250 ng of pGL3-basic MMP9 promoter full length (−923 bp to + 18 bp) or 5′-deletion MMP9 promoter constructs and 30 ng of control plasmid expressing renilla luciferase under the control of thymidine kinase promoter (pRL-TK). (**B**) Results were expressed as fold increase in RLU over the control normalized to pGL3-basic MMP9 expression. 5′-deletion constructs demonstrated significant increases in luciferase activity within the −366 to −216 promoter region of the MMP9 promoter. (**C**,**D**) EBS-MUT1/2/5 sites displayed significant reductions in luciferase activity compared to the full length −366 bp-MMP9-pGL3 promoter plasmid. (**E**) Activity of the—366 bp-MMP9-pGL3 ETS1 binding site mutants (EBS-MUT 1, 2, 1 + 2, and 5) were further investigated by transient transfection into A253 (n = 6), iSGEC-nSS2 (n = 6), and iSGEC-pSS1 (n = 4). (**F**) ChIP-qPCR assay performed with SGCLs and iSGECs was utilized to assess the functional relevance of ETS1 interaction with the MMP9 promoter spanning across EBSMUT1,2, and 5. Cell lysates immunoprecipitated with normal mouse IgG (negative control) (blue) or ETS1 (red) antibodies. Samples were normalized to 5% of sample input. Significant comparisons among control and ETS1 antibody were made by Mann–Whitney U-test with p values indicated over their respective bars (***p < 0.001), (**p < 0.01), (*p < 0.05). Error bars represent mean +/− standard deviation (SD).

We determined the most responsive regions of the MMP9 promoter spanning several putative ETS-1 binding sites (EBS) by transient transfection of the ETS1 overexpressing clone, HMC-3A-E6. The most responsive region was determined to span −216 bp to −366 bp upstream of MMP9 transcription start site (TSS) (Fig. 3A,B). The largest increase in luciferase activity was observed for this region with ~ twofold higher activity than the −216 bp and ~ 2.5-fold over the −439 bp truncates. Two putative EBSs within this promoter region (−216 bp to −366 bp) were uncovered using the online ALGGEN-PROMO tool. Based on consensus ETS1 binding sequence 5″-GGAA/(T)-3″, we selected an additional three putative EBS within this region (Fig. 3C). Site-directed mutagenesis revealed three separate sites responsible for ETS1 binding on MMP9 promoter (Fig. 3C,D). EBS-MUT1 contains the sequence 5′-AAGGGAT-3′ with the ETS1 consensus sequence underlined. EBS-MUT2 contains the sequence 5′-GGATCC-3′ sites, conferring two potential binding sites in a palindromic orientation. Mutations disrupting MUT1 or MUT2 regions reduced promoter activity by (57%) −2.32-fold and (59%) −2.44-fold, respectively.

To confirm whether the presence of one or both sites was required for ETS1 regulatory activity, we constructed the −366 bp-MMP9 pGL3 EBS-MUT1 + 2 plasmid containing both EBS-MUT1 and EBS-MUT2 site mutations. Changes to both sites demonstrated either site is required individually for ETS1 activity, whereas tandem mutations did not further decrease MMP9 promoter activity (66%) (−2.93-fold) (p > 0.05) compared to either EBS-MUT1 or EBS-MUT2 individually. The final EBS (MUT5) represents a consensus EBS previously described as a significant motif in ETS1-responsive promoter regions of both ETS/AP-1 responsive RAS/ERK mediated epithelial gene expression and B-cell maturation^29,32^. Here, EBS-MUT5 (5′-CAGGAAA-3″) reduced promoter activity by 70%, i.e., ~ 3.3-fold, compared to the full length −366 bp-MMP9 pGL3 plasmid (p < 0.01).

Serial deletion and site-directed mutagenesis of the −216 bp to −366 bp MMP9 promoter region revealed three potential EBSs, that were further analyzed in A253, iSGEC-pSS1, and iSGEC-nSS2 cells (Fig. 3E). iSGEC-nSS2 and A253 responded similarly to HMC-3A (E6) when transfected with EBS-MUT1, 2 or MUT5, where significant reductions in luciferase activity, i.e., 60%/47%/62% and 43%/49%/58% were observed, respectively. Overall, iSGEC-pSS1 showed a modest decrease in MMP9 promoter expression for all EBS-MUTS (1, 2, 1 + 2, and 5) with the largest decrease observed in EBS-MUT1 (−1.3-fold) (24%) (p > 0.05).

To confirm ETS1 was responsible for binding to the MMP9 promoter region within −216 bp to −366 bp of the TSS, ChIP was performed using an ETS1 antibody with qPCR primers targeting the 224 bp surrounding region from −421 bp to −197 bp of the MMP9 TSS (Fig. 3F). All four cell lines displayed a significant increase in the percentage of input bound over the control (normal mouse IgG). Together, these results demonstrated the regulation of ETS1 at either the EBS-MUT 1, 2, 1 + 2, and/or 5 site(s) was responsible for MMP9 promoter activation in the tested salivary gland epithelial cell lines.

### Phos(T38)-ETS1 nuclear localization and MMP9 expression in iSGECs

**Figure 4:**
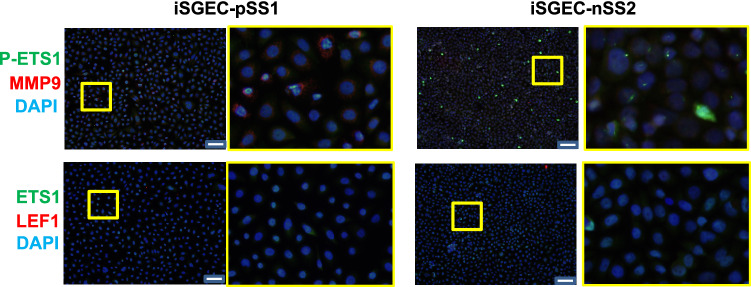
Characterization of epithelial and progenitor cell markers in iSGECs by immunofluorescence. iSGEC-pSS1 (left) and iSGEC-nSS2 (right) were cultured on gelatin coated 8-well tissue culture slides for 48–72 h. Phosphorylated ETS1 (Thr38) (P-ETS1) displayed greater overall nuclear localization and intensity in iSGEC-nSS2 and was co-expressed with increased MMP9 levels in iSGEC-pSS1. ETS1 and LEF1 demonstrated relatively low expression levels in iSGECs when assessed by IF. iSGEC-nSS2 cells expressed Phos(T38)-ETS1 uniformly as small nuclear bodies interspersed with high- expressing whole-cell localized Phos(T38)-ETS1, regardless of MMP9 levels. The overall intensity and localization of ETS1 and LEF1 was unremarkable in either cell line or highlighted obvious differences in some cells. Representative regions (yellow) are highlighted and displayed adjacent their respective image. Images were captured at 20 × magnification and white scale bars represent 100 μm.

The regulatory role of ETS1 in MMP9 expression was markedly different in non-pSS sicca *vs.* pSS derived iSGECs. To further characterize the mechanism(s) contributing to the regulatory disparities of ETS1 and possible relationship to pSS pathogenesis, we analyzed the basal expression of ETS1, Phos(T38)-ETS1 and MMP9 by immunofluorescence assay (IF). Dual IF using a specific Phos(T38)-ETS1 antibody revealed a distinct relationship among nuclear Phos(T38)-ETS1 and MMP9 expression by immunocytochemistry (Fig. 4) in iSGEC-pSS1 cells.

### Effects of ETS1 overexpression in iSGECs on epithelial and EMT-associated markers

We assessed whether ETS1 overexpression altered the expression of salivary progenitor cell (EpCAM, K5), epithelial (CDH1, AQP5, β-catenin), mesenchymal (VIM), and MMP9 markers in whole cell lysates of iSGECs by transfection with ETS1 expression vectors. MMP9 protein expression increased after ETS1 overexpression in both iSGEC lines (Supplemental Fig. 3). EMT-associated markers, CDH1 and VIM displayed relative changes after ETS1 overexpression, where CDH1 expression decreased as VIM increased in iSGEC-nSS2 (Supplemental Fig. 3). Conversely, iSGEC-pSS1 levels of CDH1 remained relatively unchanged while VIM expression increased after ETS1 overexpression. Lastly, the expression of AQP5 and EpCAM increased in iSGEC-nSS2 cells after transfection with ETS1, which was not observed in iSGEC-pSS1.

## Discussion

We demonstrated for the first time the overexpression of ETS1 and MMP9 in cultured SGECs of pSS patients and confirmed a significant relationship between ETS1 and MMP9 expression. We utilized siRNA targeting ETS1 in two SGCLs (A253 and HMC-3A) and two iSGEC lines (iSGEC-pSS1 and iSGEC-nSS2), which led to a reduction in both MMP9 mRNA and protein levels. To uncover the regulatory nature of this connection, we generated an ETS1 overexpressing clone of the SGCL HMC-3A to investigate ETS-mediated regulation of MMP9. Luciferase activity of the MMP9 promoter implied significant ETS1 regulatory binding features within the −216 bp to −366 bp upstream region. Site-directed mutagenesis of three binding sites demonstrated that ETS1 regulated MMP9 transcription in all four cell lines tested, which was further confirmed by ChIP assay.

### Consequences of pathologic MMP9 overexpression in the salivary epithelium

The functional role of MMP9 in the pre-immune phase of pSS has so far to be fully elucidated. Supplementary Fig. 2 presents a model outlining the downstream effects of MMP9 overexpression and possible consequences of ECM breakdown, altogether consistent with pathological observations demonstrated in pSS salivary glands. It was previously shown that pSS mouse models demonstrate breakdown of glandular structures by MMP9 in the epithelium, early in disease onset with limited or no presence of infiltrating lymphocytes^13^. Notably, MMP9 has also been shown to be detrimental to lacrimal glands in murine models of Sjӧgren’s Syndrome^43^. Our study establishes ETS1 as a driver of the pathologic MMP9 overexpression in the salivary epithelium of both pSS and non-pSS SGECs (0 < FS < 1). pSS pathogenesis involves a pre-inflammatory and immune phase, where the disorganization, breakdown, and reduced secretion (pre-inflammatory) of the salivary glands is exacerbated by lymphocytic infiltration (immune phase)^44^. Although the current classification criteria for pSS includes multiple clinical observations and a single assessment of LSG infiltrates (i.e., focal scoring) when negative for serum Anti-SSA, pre-immune pSS has significant value in preventive therapy development^6^. pSS SGECs expressed MMP9 at higher levels compared to the non-pSS (FS = 0) group and is consistent with the in vivo* disease* pathology (Fig. 1A). Moreover, non-pSS (0 < FS < 1) SGECs exhibited a similar increase in both MMP9 and ETS1 as the pSS FS ≥ 1 group, which may represent a state of sicca progression towards pSS.

### Differences in ETS1 activity on the MMP9 promoter in iSGEC-nSS2 vs. -pSS1

The regulatory impact of ETS1 on MMP9 expression was present but reduced within the iSGEC-pSS1 cells when compared to iSGEC-nSS2 (intermediate (FS = 0.16), non-pSS sicca) (Figs. 1E, 2E, and 3E). This unanticipated observation might explain some of the discrepancies observed regarding MMP9 expression and its regulation in the pSS salivary gland epithelium^26,45,46^. Transcriptional regulation of MMP9 is likely more dependent on ETS1 in sicca patients (non-pSS, 0 < FS < 1) when inflammation is limited. As the disease shifts to the immune-phase with lymphocytic infiltrates, other MMP9 regulators may play a role, such as the inflammatory cytokine TNF-α or transcription factor NF-κβ^45,46^. The presence of other factors mediating ETS1 binding could explain the smaller reduction in MMP9 promoter activity of iSGEC-pSS1 (Fig. 3E), although significant promoter binding by ETS1 was still observed (Fig. 3F).

### Modulation of ETS1 activity and function by cofactors or post-translational modification

The function and activity of ETS1 has been demonstrated to be heavily dependent on cooperative binding partners^29,32^. ETS1 activity is further modulated by its phosphorylation status at different sites, such as ERK1/2 mediated Thr 38/Thr 72^29,32^. The ERK1/2 pathway has been implicated in pSS SGEC cytokine production and EMT-mediated fibrosis of salivary gland tissue ^47,48^. Post-translational modifications to ETS1 alongside cooperative binding partners could explain the difference in iSGEC-nSS2 and iSGEC-pSS1 MMP9 expression (Figs. 1E,F,2E).

### ETS1 alters the expression of EMT associated proteins

The effects of ETS1 on EMT associated proteins by Western blot (Supplemental Fig. 3) demonstrated an impact on epithelial/mesenchymal gene expression. ETS1enhanced VIM expression in iSGEC-pSS1, whereas ETS1 had little to no effect on VIM expression iSGEC-nSS2. These results are consistent with previous studies highlighting the role of ETS1 in EMT where ETS1 itself was unable to induce EMT but potentiated and maintained the cells in an EMT-like state^49^. iSGEC-pSS1 cells expressed VIM at a higher basal level than iSGEC-nSS2 cells and similarly responded greater to ETS1 overexpression. Despite the differences in basal expression of EMT-associated markers, both cell lines maintained consistent K5 expression after ETS1 transfection. K5 expression is a characteristic of progenitor cells derived from the basal epithelium and appears to be independent of ETS1^50^.

### Caveats/pitfalls

It is important to note we did not assess the downstream effects of MMP9 inhibition by ETS1 such as MMP9-mediated downregulation of CXCL10 under IFN-γ stimulation^45^. MMP9 has been previously demonstrated to mediate EMT in cell culture models^51^. However, the relationship between MMP9 and EMT-related genes within the salivary epithelium was not explored within this study. Additionally, we did not address possible mechanisms governing the overexpression of ETS1 within the epithelium of pSS patients. Interestingly, LINE-1 ORF-1p, a retrotransposon element typically silenced through DNA methylation is overexpressed in pSS patients, interacts with ETS1 increasing nuclear concentrations and facilitates ETS1-DNA binding^52,53^. The series of etiologic events contributing to pSS is not well understood, but some epigenetic changes such as hypomethylation have been reported as a potential causative agent^52,54^. Potential sources of non-immunologic LINE-1 ORF-1p hypomethylation could be due to improper X-chromosome inactivation where genes controlling methylation located on the X-chromosome are improperly silenced, leading to global methylation changes over time and an X-chromosome dose-effect^54^.

## Conclusion

In conclusion, we have shown that ETS1 is able to upregulate MMP9 expression in non-pSS sicca derived iSGECs and to a lesser extent in pSS derived iSGECs. Additionally, we found differences in the expression of EMT factors possibly contributing to fibrosis of the salivary gland. Also, differences in MMP9 regulation might reflect progression of the salivary gland ECM destruction towards a pro-inflammatory stage. Using both non-pSS and pSS iSGEC cell culture models, ETS1 was determined to bind at three separate locations on the MMP9 promoter, providing a non-immunologic mediated mechanism of MMP9 expression *in-vitro.* Investigating the mechanisms governing ETS1 expression promoting pSS pathogenesis through MMP9 upregulation could provide new therapeutic targets to reduce salivary gland degradation and improve acinar function.

## Supplementary Information

Supplementary Information.

## Data Availability

The datasets generated and/or analyzed during the current study are available in the GitHub repository https://github.com/mbeckm01.
