# Supplementary material for: Regulation of MMP9 transcription by ETS1 in immortalized salivary gland epithelial cells of patients with salivary hypofunction and primary Sjögren’s syndrome
Source: Sci Rep. 2022 Aug 25;12:14552. doi: 10.1038/s41598-022-18576-z (PMC9411565; doi:10.1038/s41598-022-18576-z)
Supplement: Supplementary file 1 — Supplementary Information. [file 41598_2022_18576_MOESM1_ESM.pdf]

Supplemental Figure 1: Experimental workflow for determination of regulation of MMP9 expression by transcription factor ETS1

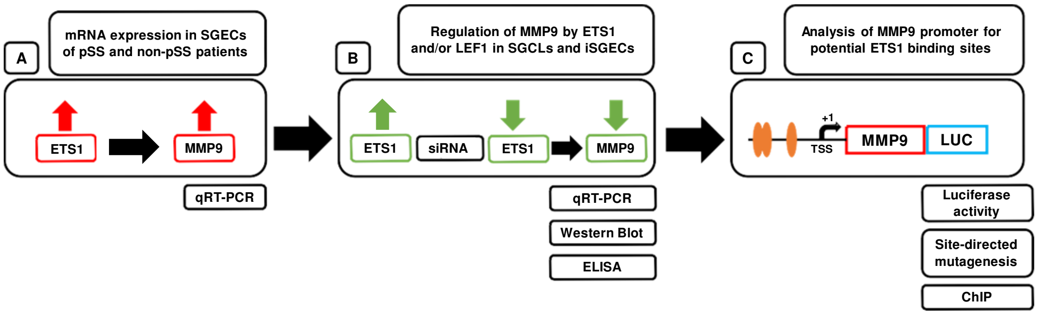

Legend. Experimental strategy and assays used to determine ETS1 regulation of the MMP9 promoter in the pSS salivary gland epithelial cells. We have previously shown that transcription factors ETS1 is overexpressed in the epithelium of pSS labial salivary gland biopsies and that their expression correlates with MMP9 protein expression. Previous studies have demonstrated regulatory roles for ETS1 in controlling MMP9 expression in T-Cells, however, their relationship to MMP9 expression has yet to be investigated in pSS epithelium. **(A)** We first investigated whether the overexpression of ETS1 and MMP9 persisted *in-vitro* using SGEC explant cultures from LSG biopsies of pSS and non-pSS patients by qRT-PCR. **(B)** Due to the transient nature of primary SGEC cultures, two SGCLs HMC-3A, A253 of female and male origin, respectively, and two iSGEC lines of female origin were utilized as models to investigate the regulatory relationship between ETS1 and MMP9 at both mRNA and protein levels by siRNA knockdown. **(C)** To investigate regulation of MMP9 further, we generated an ETS1 overexpressing SGCL clone of HMC-3A to initially assess the direct transcriptional activity and binding of ETS1 on the MMP9 promoter. The MMP9 promoter region (-914bp to +18bp) was inserted into a luciferase reporter plasmid to identify the significant DNA regulatory regions. These regions were narrowed down to individual ETS1 binding sites by a site directed mutagenesis approach. Lastly, the *in-vitro* binding of ETS1 onto the MMP9 promoter was confirmed by ChIP-qPCR on both iSGECs and SGCLs models.

SGEC= Salivary gland epithelial cell; LSG= Labial salivary gland; pSS= Primary Sjögren's syndrome (Focus Score ≥1); non-pSS= non-primary Sjögren's syndrome (*i.e.*, Focus Score <1); SGCLs= salivary gland cancer cell lines represented by HMC-3A (human mucoepidermoid carcinoma 3A) and A253 (submaxillary salivary gland carcinoma); iSGEC= immortalized salivary gland epithelial cell line (*i.e.*, iSGEC-pSS1, iSGEC-nSS2); ChIP= Chromatin Immunoprecipitation; qRT-PCR; quantitative real-time polymerase chain reaction; LUC= Luciferase; MMP9= Matrix metallopeptidase 9; ETS1= V-Ets Avian Erythroblastosis Virus E26 Oncogene Homolog 1; TSS= Transcription Start Site; ELISA= Enzyme-linked immunosorbent assay.

Supplemental Figure 2: Proposed model for ETS1 mediated regulation of MMP9 expression and downstream effects of ECM degradation observed in pSS salivary and lacrimal glands

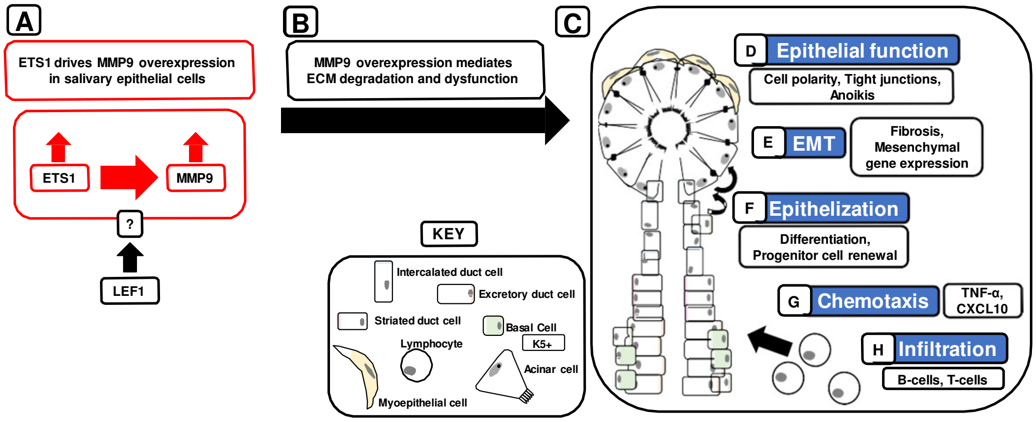

Legend. (A) Our *in vitro* experimental results presented in this study and previous work on salivary gland biopsies provide evidence that ETS1 regulates MMP9 overexpression in the salivary gland epithelium of non-pSS and pSS patients. **(B)** The overexpression of MMP9 within the salivary epithelium is a known pathological marker in pSS salivary gland dysfunction and is mainly driven by ETS1. **(C)** MMP9 targets a variety of ECM components with significant roles in maintaining glandular epithelial homeostasis and function. **(D)** MMP9 degrades tight junctions (cell-cell) in salivary and lacrimal glands, alongside hemi-desmosomes (ECM-cell) within salivary glands, which disrupts epithelial cell polarity and directional secretion by acinar cells. Loss of ECM-cell connections leads to apoptosis of acinar cells through anoikis. **(E)** Disruption of ECM-cell connections within the epithelium promotes EMT and fibrosis. **(F)** Furthermore, re-epithelization and differentiation of resident progenitor cells rely on ECM components to direct movement and activity. Additionally, acinar cells rely on collagen for differentiation. The overabundance of $K5^{+}$ progenitor cells, which normally can differentiate into both acinar and ductal cells during injury and stress, reflects the lack of a proper re- epithelization process occurring in pSS salivary glands, which is driven by MMP9 overexpression, thereby preventing ECM maturation. **(G)** MMP9 enhances chemotaxis and **(H)** infiltration of lymphocytes into both salivary and lacrimal glandular tissue by degrading the ECM to facilitate lymphocyte migration. Meanwhile, cleavage-activation of variety of secreted and ECM-bound chemokines (CXCL10, TNFα) leads to increased inflammation.

ECM= extracellular matrix; EMT= epithelial-mesenchymal transition; K5= Cytokeratin 5; CXCL10= C-X-C motif chemokine ligand 10; TNFα= Tumor necrosis factor α
SGEC= Salivary gland epithelial cell; pSS= Primary Sjögren's syndrome (Focus Score ≥1); MMP9= Matrix metallopeptidase 9; ETS1= V-Ets Avian Erythroblastosis Virus E26 Oncogene Homolog 1.

Supplemental Figure 3: Effects of ETS1 protein expression of epithelial and mesenchymal cell markers in iSGECs

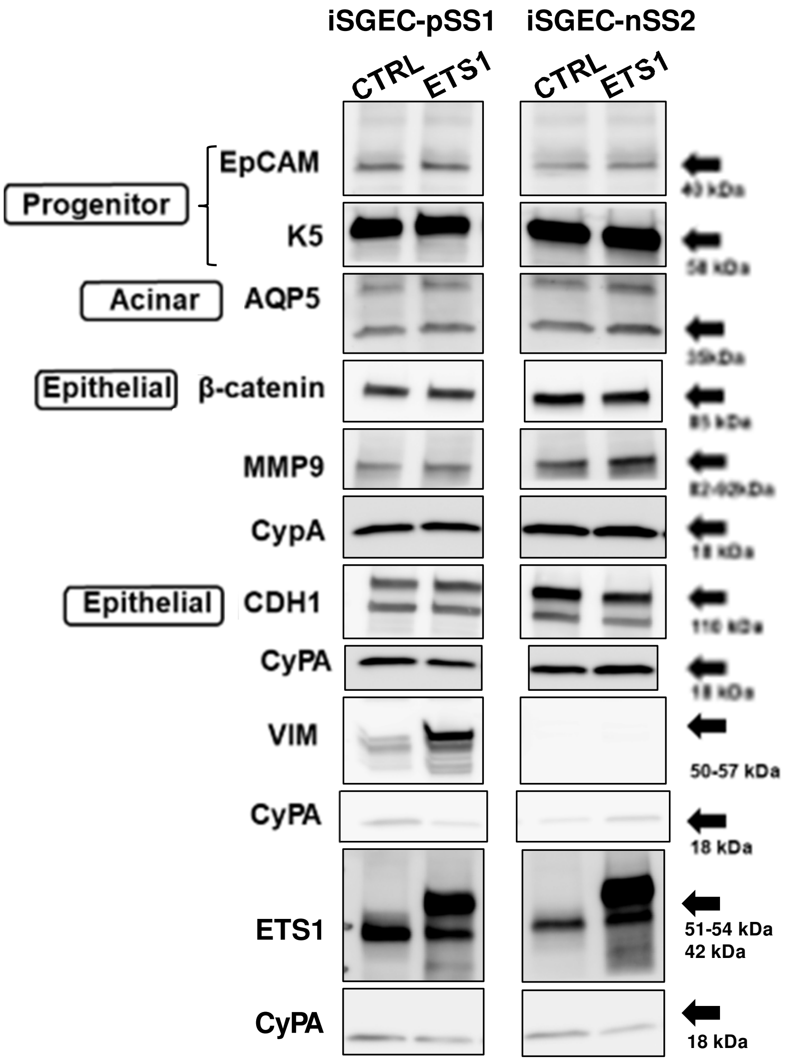

Legend. iSGECs were transiently transfected with 500ng of pCMV3 overexpressing plasmids and after 72hrs whole cell protein lysates were collected for Western blot analysis. Changes in the expression of progenitor cell markers EpCAM and K5, EMT epithelial markers CDH1 and β-catenin and mesenchymal VIM, and acinar AQP5 after overexpression of ETS1 were determined. Each lane was loaded with equal amounts of protein and Cyclophilin A (CyPA) used to compare loading among the experimental (pCMV3-ETS1) and pCMV3-empty (CTRL). Expected protein sizes are listed under their respective arrow indicating the correct band. Protein targets are grouped according to their corresponding loading control (CyPA) ran on the same gel/blot. iSGECs exhibited differing levels of endogenous epithelial and mesenchymal markers with iSGEC-pSS1 displaying a greater mesenchymal-like profile. VIM expression was increased by ETS1 in iSGEC-pSS1 but not in iSGEC-nSS2. In contrast, the overexpression of ETS1 reduced epithelial marker CDH1 expression in iSGEC- nSS2. AQP5 is aquaporin 5; CDH1 is cadherin 1; EpCAM is epithelial cell adhesion molecule; K5 is keratin 5; VIM is vimentin.

Supplemental Table 1: Primers

Target	Primer Sequence
ETS1-F	5’-CTGCGCCCTGGGTAAAGA-3’
ETS1-R	5’-ACATCCTCTTTCTGCAGGATCT-3’
MMP9-F	5’-CCTGGGCAGATTCCAAACCT-3’
MMP9-R	5’-GCAAGTCTTCCGAGTAGTTTTGGAT-3’
GAPDH-F	5’-AGGGCTGCTTTTAACTCTGGT-3’
GAPDH-R	5’-CCCCACTTGATTTTGGAGGGA-3’
MMP9 CHIP-F	5’-AGGGGGATCATTAGTTTCAGAAA -3’
MMP9 CHIP-R	5’-ACTGCCAAGTCAGGCAAG-3’
-366bp-EBS-MUT1-F	5’-ACCAAG**TT**ATGGGGGATCCCTCCAGCTTC-3’
-366bp-EBS-MUT1-R	5’-CCCCCATAACTTGGTCTGAAAGCCTCCAGTGG-3’
-366bp-EBS-MUT2-F	5’- ATGGG**T**GAT**AA**CTCCAGCTTCATCCCCCTC-3’
-366bp-EBS-MUT2-R	5’- GGAGTTATCACCCATCCCTTGGTCTGAAAGC-3’
-366bp-EBS-MUT5-F	5’- TGCGGT**CGGG**TGCGGGTCTGGGGTCTTGC -3’
-366bp-EBS-MUT5-R	5’- CCGCACCCGACCGCAGGCCCTCAGGGG -3’

Primers used for analyzing the expression of ETS1 and MMP9 by qRT-PCR in SGECs, SGCLs, and iSGECs. The primers for the MMP9 promoter putative ETS1 binding sites are listed. Significant primers for ETS1 binding site mutagenesis to generate EBS-MUT constructs within the -366bp MMP9 promoter luciferase are listed. Nucleotide sequences altered within the 5’-GGAA/T-3’ EBS binding motif of the constructs are shown in bold. Sanger sequencing and restriction digestion were performed to confirm mutagenesis.

Supplemental Table 2: Primary and Secondary Antibodies

Target	Assay	Species	Source	Cat#	Dilution
ETS1	WB	Mouse	SCBT	sc-55581	1:250
MMP9	WB, IF	Mouse	SCBT	sc-393859	1:200,1:100
ETS1	IF	Rabbit	Cell Signaling	14069S	1:200
Phospho-ETS1 (Thr38)	IF	Rabbit	Invitrogen	PA5-37572	1:200
EpCAM	WB	Mouse	eBioscience	14-9326-82	1:400
K5	WB	Rabbit	Biolegend	905504	1:500
AQP5	WB	Mouse	SCBT	sc-514022	1:100
E-Cadherin	WB	Rabbit	Proteintech	20874-1-AP	1:400
B-Catenin	WB	Mouse	SCBT	sc-7963	1:300
Vimentin	WB	Mouse	SCBT	sc-6260	1:500
Cofilin	WB	Mouse	SCBT	sc-376476	1:1000
CyPA	WB	Mouse	SCBT	sc-134310	1:1000
Anti-mouse IgG-HRP	WB	Mouse	Cell Signaling	7076	1:2000
Anti-rabbit IgG-HRP	WB	Rabbit	Cell Signaling	7074	1:2000
Goat anti-rabbit IgG-Alexa-488	IF	Rabbit	Invitrogen	A32731	1:400
Goat anti-mouse IgG-Alexa-594	IF	Mouse	Invitrogen	A32742	1:400

Primary and secondary antibodies with their associated dilutions used for either Western blotting or immunofluorescence assays. Primary antibodies were diluted in TBS with 2% BSA, 0.1% Tween-20, and 0.05% sodium azide. Secondary antibodies were diluted in DPBS with 0.1% Tween-20. WB= Western Blot; IF= immunofluorescence

Supplemental Table 3: Clinical characteristics of patients who provided salivary gland biopsy tissue to derive primary SGECs cultures

Patient	Focus Score	Diagnosis	Anti SSA+	Race	Sex	Age	Unstim. Salivary Flow (mL/min)	Schirmer's (+/-)
Pt1	0	no support for SS; no mention of chronic sialadenitis	negative	c	f	69	0	negative
Pt2	0	no support for SS; mild focal chronic sialadenitis	negative	c	f	37	0.104	positive
Pt3	0	no support for SS; mild chronic sclerosing sialadenitis	negative	b	f	52	0.126	positive
Pt4	0	no support for SS; mild non-specific sialadenitis	negative	c	f	31	0.276	positive
Pt5	0	no support for SS; nonspecific chronic sialadenitis	negative	c	f	33	0.13	negative
Pt6	0.16	no support for SS; mild focal sialadenitis	negative	c	f	47	0.004	negative
Pt7	0.3	no support for SS; Mild focal non-specific chronic sialadenitis	negative	c	f	57	0.044	not done
Pt8	0.36	no support for SS; no mention of chronic sialadenitis	negative	c	f	61	0.112	positive
Pt9	0.52	no support for SS; no mention of chronic sialadenitis	negative	c	f	78	0.37	positive
Pt10	0.7	no support for SS; mild focal chronic sialadenitis	negative	c	f	60	0.138	positive
Pt11	0.7	Not available (NA)	negative	c	f	67	0	not done
Pt12	0.8	no support for SS; no mention of chronic sialadenitis	negative	c	f	38	0.026	negative
Pt13	0.84	no support for SS	negative	c	f	72	0.046	negative
Pt14	0.9	no support for SS; multifocal lymphocytic sialadenitis	negative	c	f	52	0.062	not done
Pt15	1.1	diagnosis of SS; multifocal chronic sialadenitis	negative	c	f	46	0.032	negative
Pt16	1.1	NA	negative	c	f	48	0.0006	positive
Pt17	1.23	diagnosis of pSS; multifocal chronic sialadenitis	positive	c	f	57	0.198	negative
Pt18	1.3	diagnosis ???; no mention of chronic sialadenitis	not done	NA	f	36	0.332	negative
Pt19	1.8	diagnosis of SS; multifocal chronic sialadenitis	negative	c	f	70	0.044	not done
Pt20	1.9	diagnosis of SS; focal lymphoplasmacytic infiltrates	not done	c	f	66	0.054	negative
Pt21	2.06	NA	not done	c	f	52	0.0072	negative
Pt22	2.38	diagnosis of SS; multifocal chronic sialadenitis	positive	c	f	55	0.142	negative
Pt23	3.52	diagnosis of SS; no mention of chronic sialadenitis	negative	c	f	61	0.12	positive

Supplementary Methods

Salivary gland cell lines: HMC-3A, A253, iSGEC-nSS2, and iSGEC-pSS1

A253 cells were purchased from the ATCC and cultured in McCoys 5A media (Lonza/ Hyclone) supplemented with 10% fetal bovine serum (FBS) and 2mM L-glutamine (Corning). HMC-3A cells were cultured in DMEM (High glucose + sodium pyruvate) (Lonza/ Hyclone) supplemented with (final concentration): 10% FBS (VWR), 20ng/mL EGF (Gibco), 5µg/mL insulin (MP Bio), 400ng/mL hydrocortisone (Sigma), and 2mM L-glutamine (Corning). Cells were grown (37°C; 5% CO2) for routine passaging in T-75 flask until 80-90% confluency. Trypsinization was completed using 0.25% Trypsin+ .053mM EDTA (Corning). Cultures were serum starved for 24hrs prior experimentation. Transfection experiments were carried out in basal media for HMC-3A and iSGEC cells, whereas after the 24hr serum starvation, Human Keratinocyte Growth Supplement (HKGS) (1x) (Gibco, Catalog# S0015) was added to the A253 basal media as a source of EGF to ensure consistency among experimental cell lines and their respective growth media. Both media formulations for HMC-3A and iSGEC's contain EGF, which is known to affect MMP9 expression as assessed by O-Charoenrat, P *et al.*, (Overexpression of epidermal growth factor receptor in human head and neck squamous carcinoma cell lines correlates with matrix metalloproteinase-9 expression and in vitro invasion. (2000) Int. J. Cancer, 86: 307-317.).

Primary culture of salivary gland epithelial cells

Excess labial salivary gland (LSG) biopsy tissue was placed in basal epithelial media (1:3 DMEM/Ham's F12) supplemented with 5x antibiotic/ antimycotic (stock concentration= 100x) (Gibco), 2.5% FBS, insulin (500ng/ mL), hydrocortisone (400ng/ mL), epidermal growth factor (10ng/ mL), for transport to the laboratory. LSG tissue was washed (2x) in PBS containing 1x antibiotic/ antimycotic and minced into approximately 0.5-1mm^2 pieces with multiple fragments seeded into T-75 flask. Cell were cultured in basal media (with antibiotic/ antimycotic reduced to

1x) for approximately two weeks or until reaching 70-80% confluency. Cells were washed with DPBS and removed from the flask using 0.05% trypsin+ EDTA. Trypsin was inactivated using soybean trypsin inhibitor (1:1) and the supernatant removed by centrifugation (6min, 600xg). SGECs were transferred into T-75 collagen coated flask and media switched to EpiLife Basal media (Gibco, Catalog# MEPI500CA) supplemented with HKGS (1x) (Gibco). SGECs were cultured until 70-80% confluency with residual fibroblast removed using 0.02% EDTA in DPBS. Morphologically consistent SGEC cultures were trypsinized (0.05% trypsin+ EDTA) between passages 2-4 and transferred to collagen coated 6-well tissue culture treated plates for mRNA isolation. SGECs were routinely plated on collagen-coated coverslips for epithelial confirmation by IF expression of Cytokeratin 8/18.

siRNA knockdown and transient transfection

A253 and HMC-3A cells were plated in 6-well plates 24hrs prior transfection at a concentration of 0.3-.5 x 10^6 cells per well. siRNA was transfected at a concentration of 50nM using 3µL of Lipofectamine 3000 suspended in opti-MEM (ThermoFisher) at a volume of 200µL. The transfection mixture was added dropwise to each well containing 1.8mL of media. iSGECs were plated in 6-well plates 24-hrs prior transfection at a concentration of 8x105 cells per well. siRNA was transfected at a concentration of 25nM using 1.5µL of Lipofectamine 3000 with opti-MEM at a volume of 200µL. The transfection mixture was added dropwise to each well containing 1.8mL of media.

Western blot

Proteins were isolated using Mammalian Protein Extraction Buffer (M-PER) (Pierce) following the manufacturers' recommended protocol with the addition of protease inhibitor cocktail (Millipore-Sigma). Protein extracts were briefly sonicated, and the insoluble lysate fraction removed by centrifugation (5000xg, 5min). Samples were first measured by Bradford assay and then mixed with 6x Laemmli buffer. After boiling (5min, 95°C), equal protein concentrations were loaded into and resolved by SDS-polyacrylamide gel electrophoresis (Biorad). Proteins were transferred and visualized on nitrocellulose membranes blocked with 5% nonfat dried skim milk in TBS with 0.1% Tween-20 for 1hr at RT. Primary antibodies were added at concentrations listed in **Supplemental Table 2** and incubated overnight at 4°C. Membranes were washed (3x, 5min each) and incubated with the appropriate HRP-conjugated secondary antibody for 1hr at RT. Samples were normalized to either cofilin or cyclophilin A expression and visualized with an ImageQuant-LS4000 (GE). Densitometric measurements were acquired using Li-COR Image Studio Lite software (Ver. 5.2). Due to the overlapping sizes and cross-reactivity of antibodies, blots were frequently cut into horizontal, transverse sections corresponding to their target's size before incubation with primary antibodies. Western blot images displayed as separate sections originate from the same experiment. Furthermore, to alleviate the cross-reactivity of secondary antibodies, blots were incubated with 30% H_2O_2 for 10 min, and then washed with TBST (3x, 5min each) to inhibit residual HRP-conjugated secondary for any subsequent blotting.

Chromatin immunoprecipitation (CHIP) assays

ChIP assays were performed using the EpiQuick Tissue Chromatin Immunoprecipitation Kit (Epigentek, Catalog# P-2003) following the manufactures recommended protocol with slight modifications. Cells were fixed with 1% formaldehyde for 12min at RT. After decrosslinking, nuclear extracts were sonicated on ice (8x, 20sec, 1min rest on ice in-between each cycle) to generate roughly 200-1000bp sized fragments. Antibodies (2µg of ETS1 antibody or mouse IgG

(negative control)) were added to wells and allowed to bind for 1hr at RT before blocking with 2% BSA+ calf thymus DNA (100µg/ml) in PBS (4hrs, 4°C). Prior to immunoprecipitation, cell lysates were precleared in a single unblocked well (4hrs, 4°C). Samples were split equally among the negative control (mouse IgG) and experimental (ETS1 antibody) blocked wells for incubation overnight at 4°C. Wells were extensively washed before subsequent elution, decrosslinking, and DNA purification. qPCR was performed using standard methods with primers targeting the MMP9 promoter region from -197bp to -421bp (**Supplemental Table 1**). Experimental samples and controls (mouse IgG) were normalized to 5% of the initial input of non-immunoprecipitated DNA.

ELISA

MMP9 secretion into media was measured by QuickZYME MMP9 (Quicktime Biosciences) assay following the manufactured recommended protocol. Briefly, cells were cultured in serum free media (without HKGS) for 24hrs, washed with PBS, and replaced with complete media before subjection to siRNA knockdown using the methods previously outlined. After 72hrs media was harvested for total MMP9 quantification and normalized to total cellular protein, as determined by Bradford assay (Thermo). Experimental fold changes were determined by comparison to the non-targeting siRNA control.

Promoter truncates and site-directed mutagenesis

Preliminary putative ETS1 transcription factor binding sites on the MMP9 proximal promoter region were determined initially using the online tools ALGEN-PROMO. Mutations of five consensus ETS1 (5'-GGA/T-3') binding sites (EBS) between -216bp to -366bp: EBS-MUT1 (-336bp to -332bp), EBS-MUT2 (-329bp to -323bp), EBS-MUT1+2 (-336bp to -332bp and -329bp to -323bp), EBS-MUT3 (-313bp to -309bp), EBS-MUT4, (-288bp to -284bp), EBS-MUT5

(-233bp to -229bp) were generated using the Site Directed Mutagenesis Kit (NEB) following the manufacturers' recommended protocol. Primer sequences for mutants can be found in **Supplemental Table 1**. Mutant plasmids and constructs were verified by sanger sequencing and restriction digestion.

Detection of iSGECs protein markers by Immunofluorescence

iSGECs were cultured on 8-well chambered slides (Thermo) coated with gelatin (0.2%) for 24-48hrs prior fixation with 4% PFA in PBS (12min, RT). Fixed cells were permeabilized with 0.2% Triton X-100 in PBS supplemented with 2% BSA for 25min. Slides were subsequently blocked with 5% BSA and 0.1% Tween-20 in PBS (1hr, RT). Cells were incubated with primary antibodies overnight (4°C). Following incubation, cells were washed 3x for 15min (total) at RT in PBS and incubated with secondary antibodies conjugated to fluorescent probes (1hr, RT). Dilutions for primary and secondary antibodies are listed in **Supplemental Table 2**. Cells were mounted with fluoroshield aqueous mounting media with DAPI (AbCAM) to counterstain nuclei. Slides were viewed on an Olympus BX51 fluorescence microscope (Shinjuku City, Tokyo, Japan) and photographs taken using a mounted Olympus DP70 camera (Shinjuku City, Tokyo, Japan).

10 Raw Western blot images

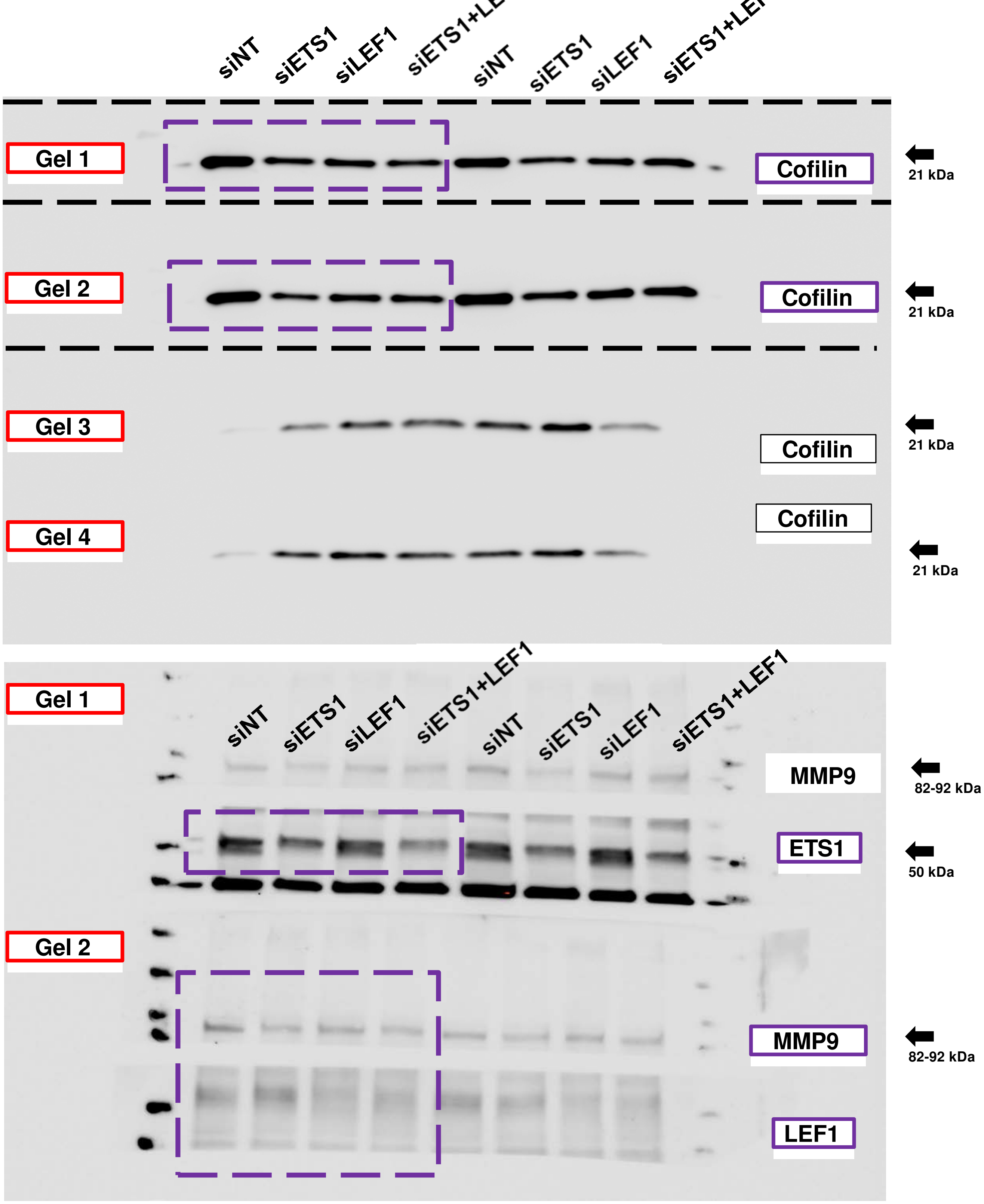

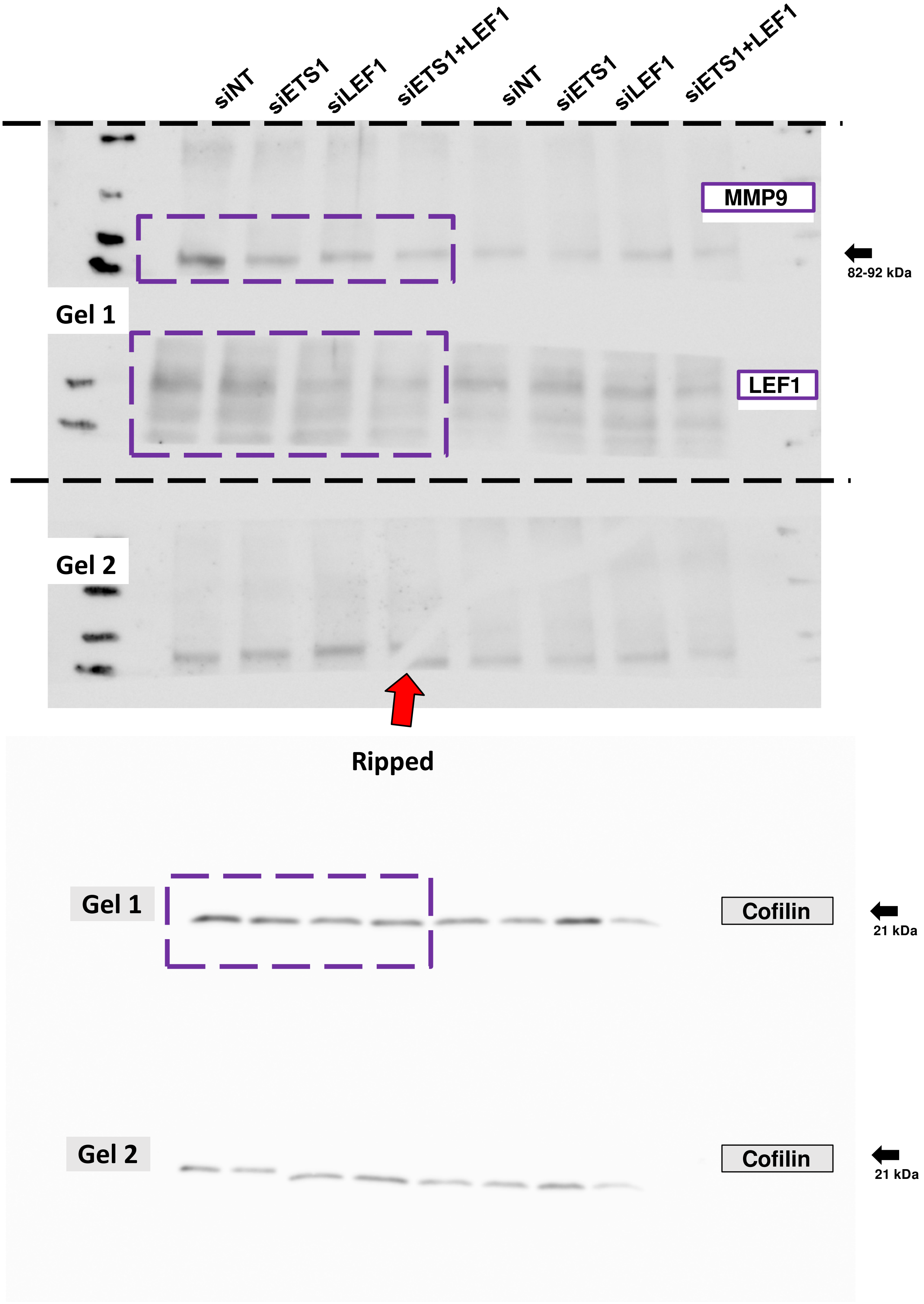
siNT
siETS1
siLEF1
siETS1+LEF1
siNT
siETS1
siLEF1
siETS1+LEF1
MMP9
82-92 kDa
Gel 1
LEF1
Gel 2
Ripped
Gel 1
Cofilin
21 kDa
Gel 2
Cofilin
21 kDa

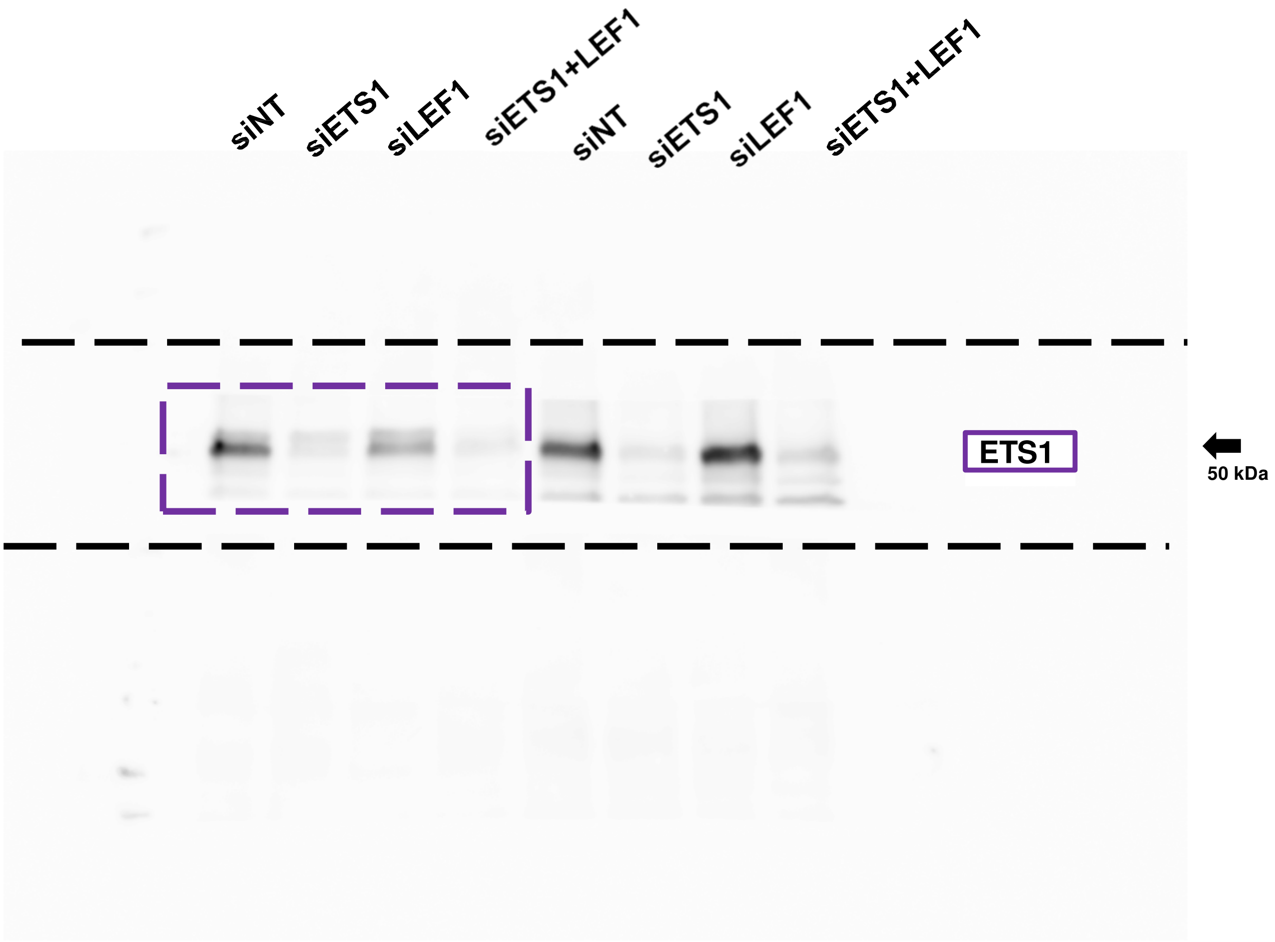
siNT
siETS1
siLEF1
siETS1+LEF1
siNT
siETS1
siLEF1
siETS1+LEF1
ETS1
50 kDa

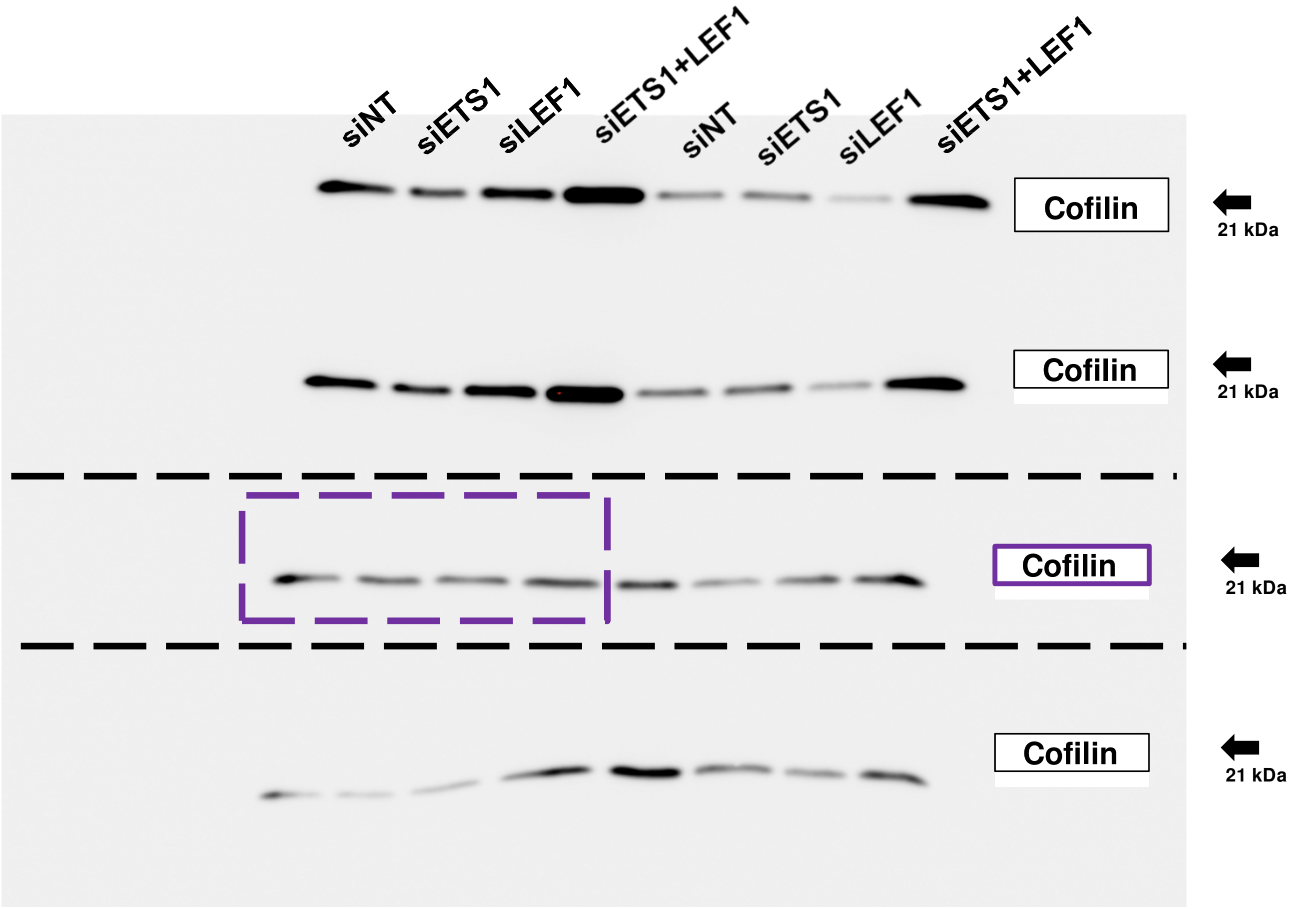
siNT
siETS1
siLEF1
siETS1+LEF1
siNT
siETS1
siLEF1
siETS1+LEF1
Cofilin
21 kDa
Cofilin
21 kDa
Cofilin
21 kDa
Cofilin
21 kDa

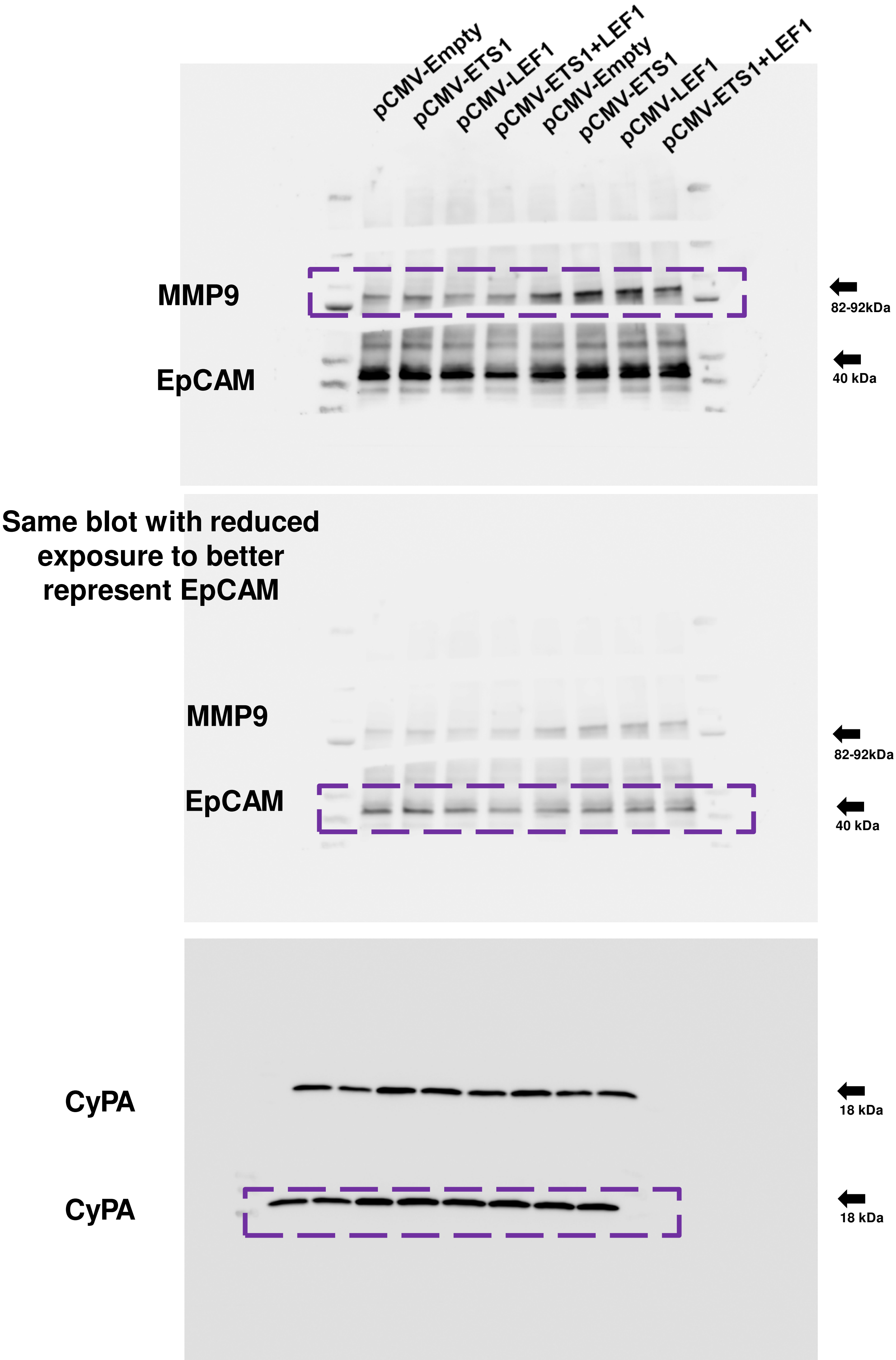

pCMV-Empty
pCMV-ETS1
pCMV-LEF1
pCMV-ETS1+LEF1
pCMV-Empty
pCMV-ETS1
pCMV-LEF1
pCMV-ETS1+LEF1
MMP9
82-92kDa
EpCAM
40 kDa
Same blot with reduced exposure to better represent EpCAM
MMP9
82-92kDa
EpCAM
40 kDa
CyPA
18 kDa
CyPA
18 kDa

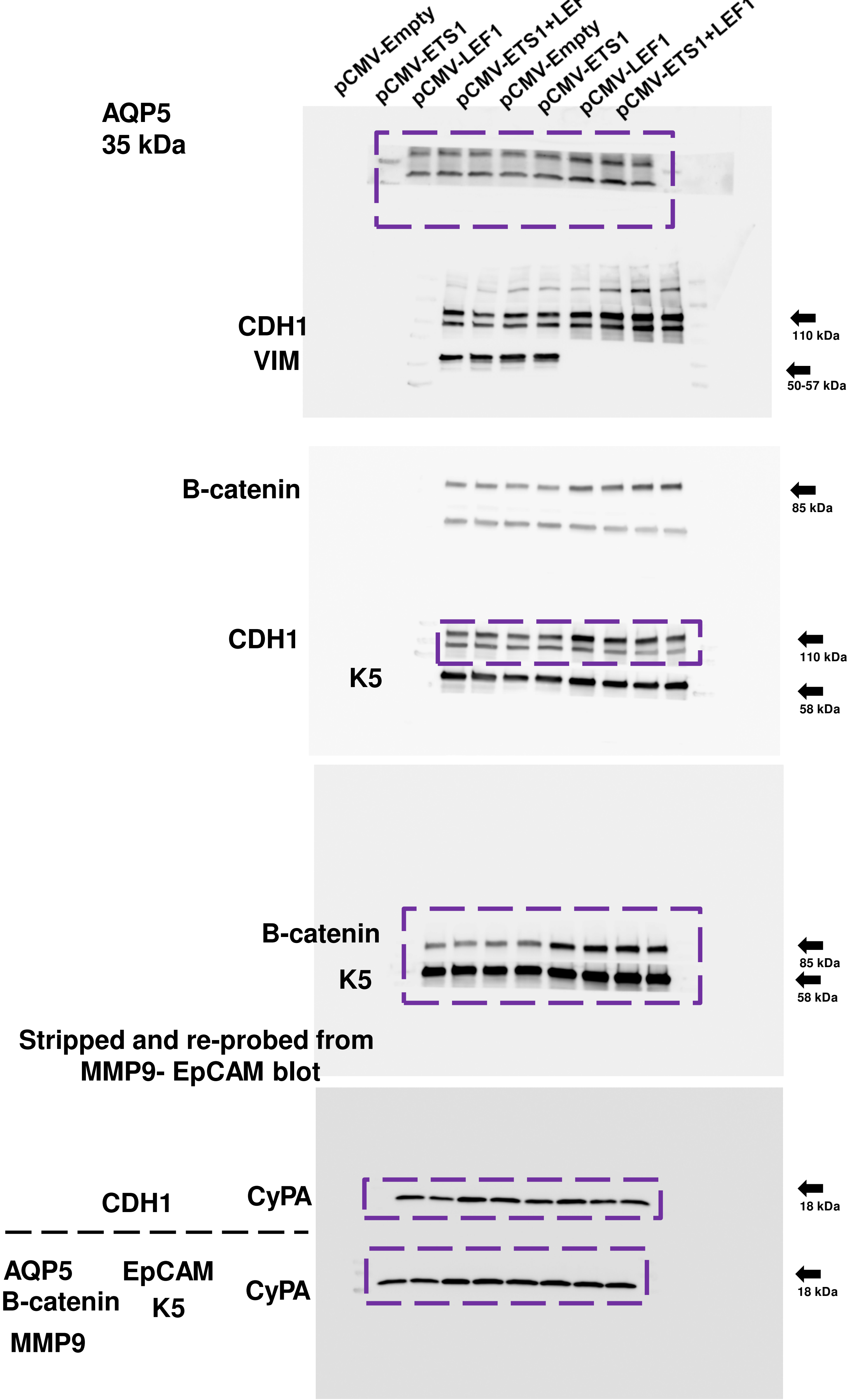
pCMV-Empty
pCMV-ETS1
pCMV-LEF1
pCMV-ETS1+LEF1
pCMV-Empty
pCMV-ETS1
pCMV-LEF1
pCMV-ETS1+LEF1
AQP5
35 kDa
CDH1
VIM
110 kDa
50-57 kDa
B-catenin
85 kDa
CDH1
K5
110 kDa
58 kDa
B-catenin
K5
85 kDa
58 kDa
Stripped and re-probed from
MMP9- EpCAM blot
CDH1
CyPA
18 kDa
AQP5
B-catenin
MMP9
EpCAM
K5
CyPA
18 kDa

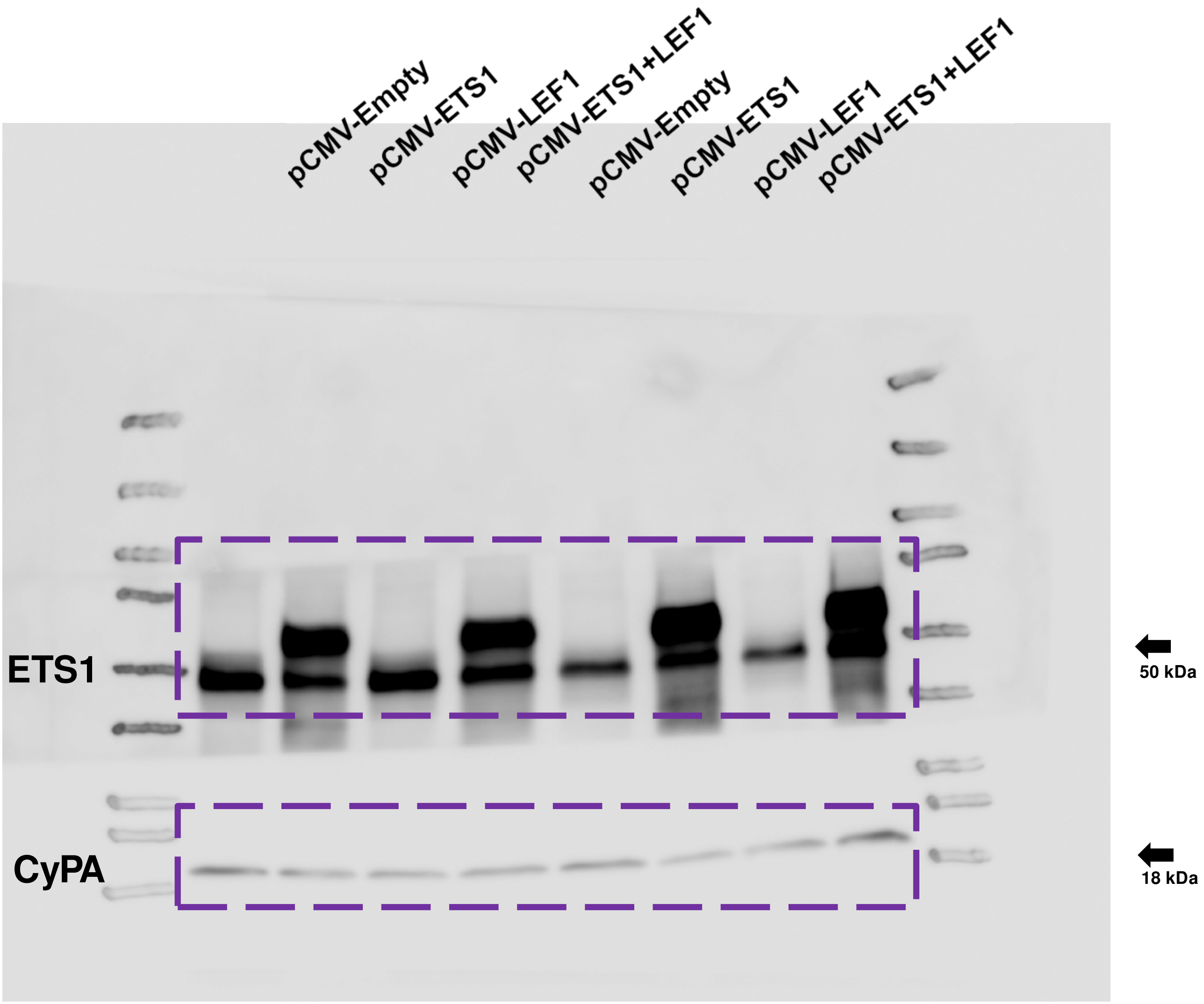
pCMV-Empty
pCMV-ETS1
pCMV-LEF1
pCMV-ETS1+LEF1
pCMV-Empty
pCMV-ETS1
pCMV-LEF1
pCMV-ETS1+LEF1
ETS1
CyPA
50 kDa
18 kDa

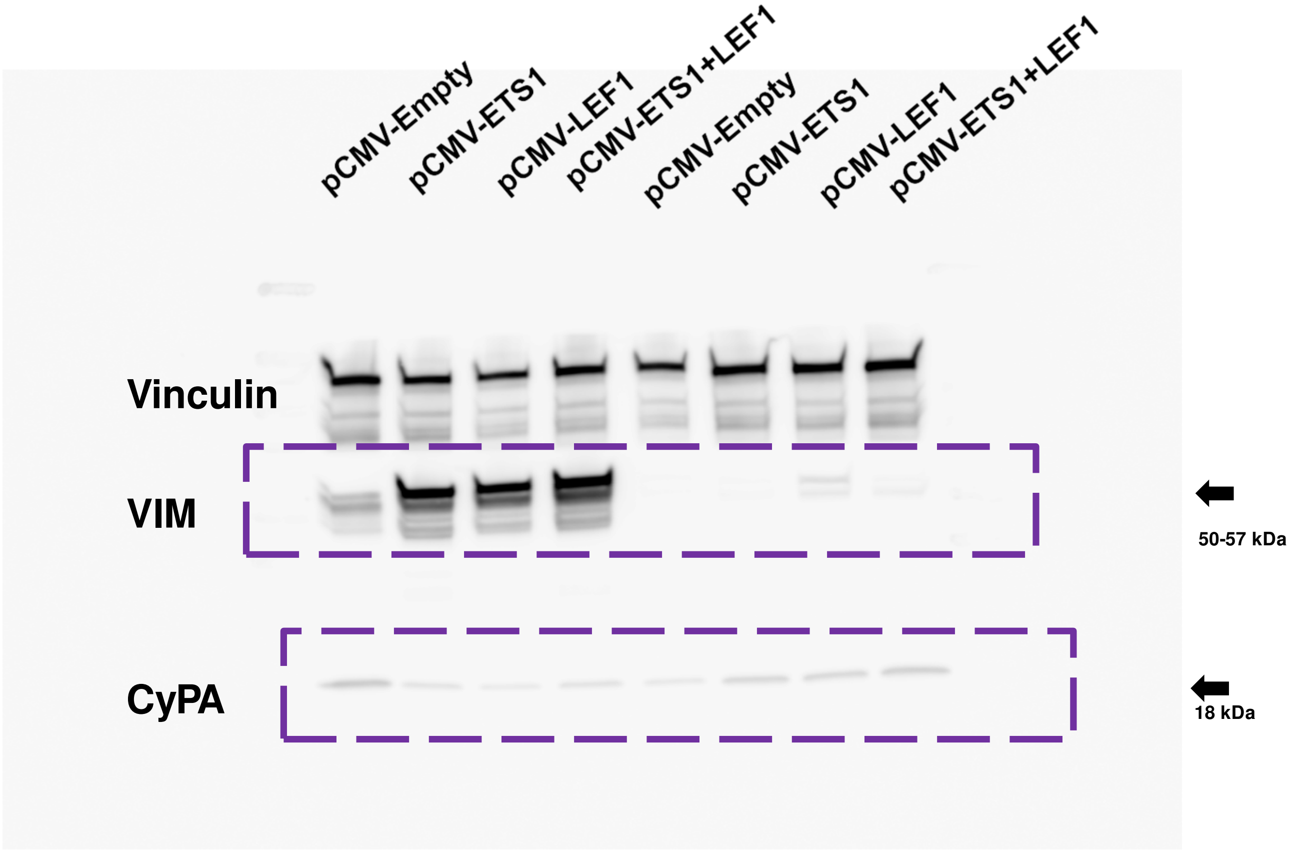
pCMV-Empty
pCMV-ETS1
pCMV-LEF1
pCMV-ETS1+LEF1
pCMV-Empty
pCMV-ETS1
pCMV-LEF1
pCMV-ETS1+LEF1
Vinculin
VIM
CyPA
50-57 kDa
18 kDa

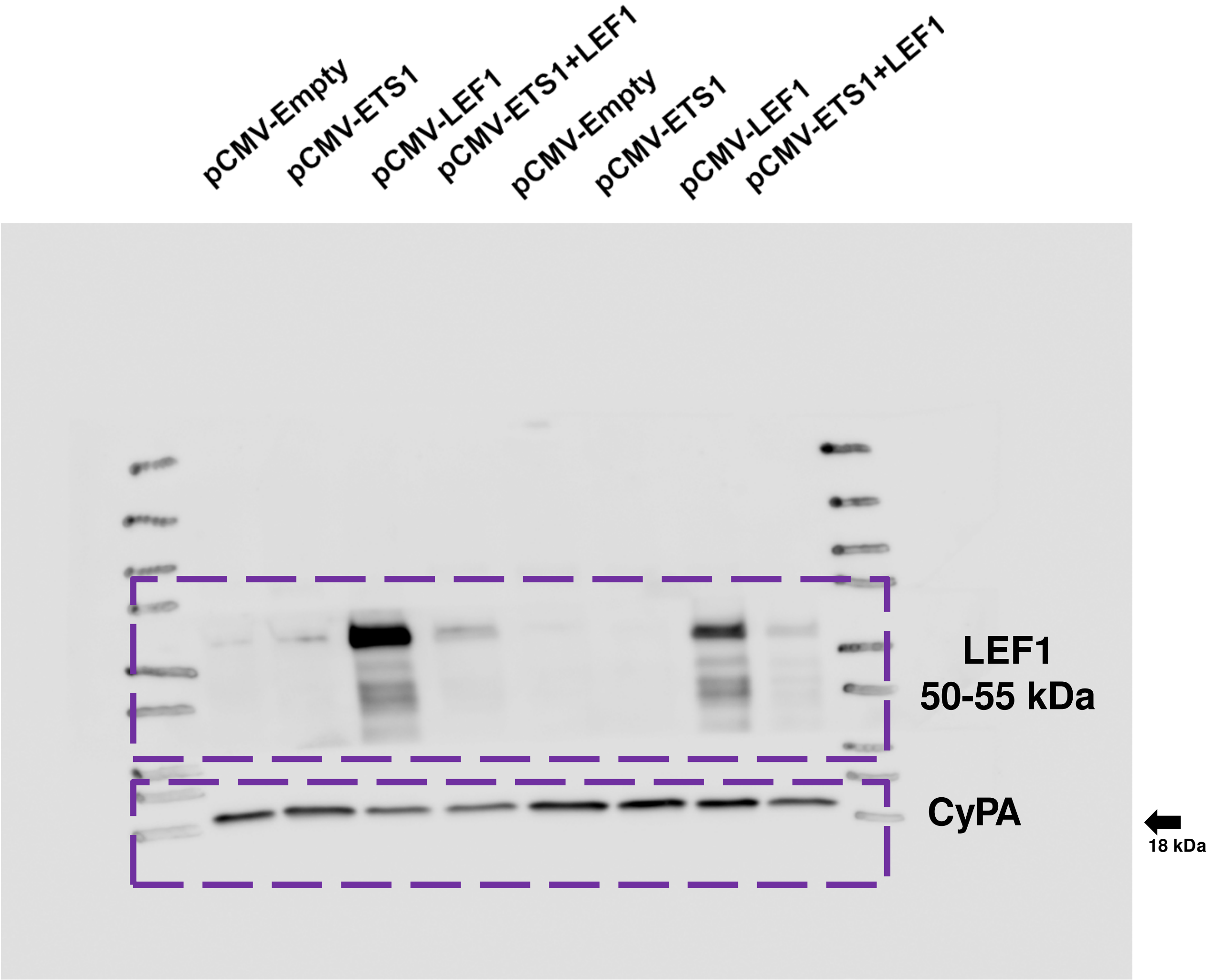
pCMV-Empty
pCMV-ETS1
pCMV-LEF1
pCMV-ETS1+LEF1
pCMV-Empty
pCMV-ETS1
pCMV-LEF1
pCMV-ETS1+LEF1
LEF1
50-55 kDa
CyPA
18 kDa
